# A 3D Human Bone and Bone Marrow‐on‐a‐Chip Model for In Vitro Bone Remodeling and Immune Cell Maintenance

**DOI:** 10.1002/advs.202518224

**Published:** 2026-07-06

**Authors:** Nina Stelzer, Melanie‐Jasmin Ort, Kristian Händler, Emely Bortel, Ioanna Maria Dimitriou, Martin Textor, Georg N. Duda, Janosch Schoon, Uwe Marx, Uwe Kornak, Annika Winter, Bernhard Hesse, Stefanie Donner, Sebastian Hardt, Oliver Klein, Simon Reinke, Malte Spielmann, Sven Geißler

**Affiliations:** ^1^ BIH‐Center for Regenerative Therapies Berlin Institute of Health at Charité‐Universitätsmedizin Berlin Berlin Germany; ^2^ Julius Wolff Institute Berlin Institute of Health at Charité‐Universitätsmedizin Berlin Berlin Germany; ^3^ Technische Universität Berlin Berlin Germany; ^4^ Institute of Medical Biochemistry Medical University Innsbruck Austria; ^5^ Institute of Human Genetics Universitätsklinikum Schleswig‐Holstein (UKSH) University of Lübeck and University of Kiel Lübeck Germany; ^6^ Xploraytion GmbH Berlin Germany; ^7^ Freie Universität Berlin Berlin Germany; ^8^ Wyss Institute for Biologically Inspired Engineering Harvard University Boston Massachusetts USA; ^9^ Center for Orthopaedics Trauma Surgery and Rehabilitation Medicine University Medicine Greifswald Greifswald Germany; ^10^ TissUse GmbH Berlin Germany; ^11^ Institute for Medical Genetics and Human Genetics Charité – Universitätsmedizin Berlin Berlin Germany; ^12^ Max Planck Institute for Molecular Genetics Berlin Germany; ^13^ Institute of Human Genetics University Medical Center Göttingen Göttingen Germany; ^14^ Center for Musculoskeletal Surgery Charité‐Universitätsmedizin Berlin Berlin Germany; ^15^ Human Molecular Genetics Group Max Planck Institute for Molecular Genetics Berlin Germany; ^16^ DZHK e.V. (German Center for Cardiovascular Research) Partner Site Hamburg/Kiel/Lübeck Germany

**Keywords:** animal alternative, bone marrow, bone remodeling, disease modeling, human microfluidic culture, organ‐on‐a‐chip, xeno‐free 3D in vitro system

## Abstract

We developed a human Bone‐on‐a‐Chip (BOAC) model that integrates autologous primary immune and bone cells on native human bone substrates within a xeno‐free, perfused environment. In contrast to existing models, which focus primarily on hematopoietic or stromal compartments, our system recapitulates key aspects of functional bone remodeling and enables the maintenance of mature immune cells (up to 42 days), with preserved functional responsiveness at defined time points during culture. Dynamic flow and sequential cell seeding facilitated osteoclast‐mediated resorption, osteoblast‐driven matrix formation, and the maintenance of donor‐specific immune profiles over extended culture periods. The balance between bone cell activity and immune cell persistence was further optimized by controlled temperature modulation. The BOAC model preserves key features of bone and bone marrow physiology, including extracellular matrix formation, soluble factor signaling, and cellular heterogeneity. The bone scaffold provides a physiologically relevant 3D architecture derived from decellularized human trabecular bone. Single‐nucleus RNA sequencing confirmed the presence of major donor‐specific immune and bone cell populations. This 3D human in vitro system provides a robust platform for translational research and personalized medicine.

## Graphical abstract

This study presents a human Bone (and Bone Marrow)‐on‐a‐Chip model based on native human bone scaffolds and autologous cells. Dynamic perfusion and sequential cell seeding replicate the physiological bone remodeling process in vitro, enabling the long‐term culture of functional, mature bone marrow immune subpopulations. The system maintains donor‐specific cellular profiles and provides a physiologically relevant platform for regenerative medicine and personalized therapies. Illustration was created with BioRender.com (https://BioRender.com/u1vlcpn).

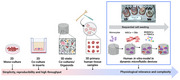

## 1 Introduction

Human bone marrow (BM) is a highly specialized organ essential for hematopoiesis and immune cell maintenance. It serves as the primary site of blood cell formation and a reservoir for lymphocytes, including memory B and T cells [1, 2, 3]. Embedded in an extracellular matrix (ECM) rich in collagen III and fibronectin, the cellular components of the BM are in close dynamic interaction with the adjacent trabecular bone. This interaction creates a specialized niche that facilitates continuous cellular crosstalk. Bone remodeling, regulated by osteoclasts, osteoblasts, and osteocytes [4], provides essential molecular signals necessary for hematopoietic stem cell differentiation and maintenance. Conversely, BM and its cellular components, including bone cell progenitors, actively modulate bone metabolism [5, 6, 7, 8]. Disrupting this interdependency can contribute to diseases such as osteoporosis, hematological malignancies, and age‐related dysfunction [9, 10].

Despite significant progress in understanding the individual functions of bone and BM, their dynamic interactions remain incompletely characterized in humans. Limited accessibility and a lack of robust methods for longitudinal studies hinder comprehensive research. Physiologically relevant in vitro bone‐on‐chip (BOAC) models represent a promising approach to investigate the complex interactions between bone and BM. Ex vivo human tissue biopsies can serve as biologically relevant templates for biomimetic bone and BM models, providing benchmarks for studying bone remodeling, immune cell function, and the release of soluble factors [11, 12]. However, their limited availability, high variability, and standardization challenges restrict their broader applicability. A promising alternative is the sequential colonization of scaffolds with different bone and BM cell types. This approach enables the controlled recapitulation of physiological conditions and flexible adaptation to specific experimental designs. In particular, decellularized human bone‐derived scaffolds provide a physiologically relevant 3D architecture resembling trabecular bone, rather than engineered biomaterials.

Early in vitro approaches for 3D bone cultures primarily focused on BM without incorporating the bone compartment [13, 14]. More recent studies have shown improvements in biological relevance. These include the ‘bone marrow‐on‐a‐chip’ [15] or hydroxyapatite‐based bone scaffolds in two‐organ‐chip systems [14], which were qualified for industrial oncology drug scheduling [16] and antibody development [17]. However, current models are still constrained in terms of cellular complexity and human relevance. Bourgine et al. hypothesized essential requirements for an ideal BM model, including a xenograft‐free 3D microarchitecture, dynamic media flow, cellular diversity, and multi‐level cell differentiation with live imaging [18]. No existing system fully satisfies these criteria, highlighting a major gap in both basic and translational bone and BM research.

Developing such a physiologically relevant in vitro bone‐BM model remains a significant challenge. While traditional 2D mono‐ and cocultures provide insights into basic cellular mechanisms, they fail to capture the intricate interactions within the bone‐BM environment [19]. Most human in vitro studies focus on osteogenesis, often neglecting its interplay with bone resorption [20]. Recent advances in 3D microfluidic cultures [21] have shifted the focus toward physiologically relevant in vitro models, where shear flow forces improve nutrient distribution, influence cell morphology, and regulate gene expression [22, 23, 24]. The impact of dynamic flow on BM immune homeostasis remains largely unexplored.

An essential aspect in 3D culture development is the selection of cell sources and scaffold colonization strategies. Primary human cells require precise isolation protocols and controlled 2D expansion to maintain their functionality. For engineering complex in vitro 3D organoids, ECM‐producing cells are crucial for establishing a supportive microenvironment and enabling adhesion of tissue cells [25, 26]. Consecutive seeding and cell density play critical roles in regulating cell behavior, nutrient diffusion, and waste removal. Yet, only very few studies have investigated optimal sequential seeding strategies for functional in vitro bone‐BM models. In addition, culture temperature is rarely discussed, even though BM has a lower temperature compared to the rest of the body [27, 28].

This study presents a physiologically relevant human 3D bone and BM model. The microfluidic system incorporates human cancellous bone scaffolds that have been sequentially colonized with different primary adult tissues and immune cells to recapitulate key aspects of bone and BM function. In this study, the decellularized bone scaffold provides a native 3D architecture of human trabecular bone. It supports the maintenance of bone turnover‐associated processes and immune cell populations in vitro, while reflecting inter‐individual variability that is relevant for translational applications. By supporting osteoclast‐mediated resorption, osteoblast‐driven matrix deposition, and immune cell viability within a single platform, it provides a versatile platform for applications in regenerative medicine and personalized therapeutic screening.

## 2 Results

We used a stepwise experimental strategy to develop a physiologically relevant in vitro bone and BM model. First, we used human femoral head tissue biopsies to define critical 3D culture parameters to preserve the immune cell compartment and enable active bone remodeling. We then refined the differentiation protocols to generate functional osteoblasts and osteoclasts from primary human cells. This involved the systematic evaluation of distinct coculture set‐ups to define parameters required for active bone turnover. These results were incorporated into a modular 3D BOAC platform with controlled flow to enable long‐term culture over defined periods of up to 42 days.

### 2.1 Dynamic Flow Improves Bone Tissue Activity and Immune Cell Maintenance During In Vitro Cultivation of Human Tissue Biopsies

**FIGURE 1:**
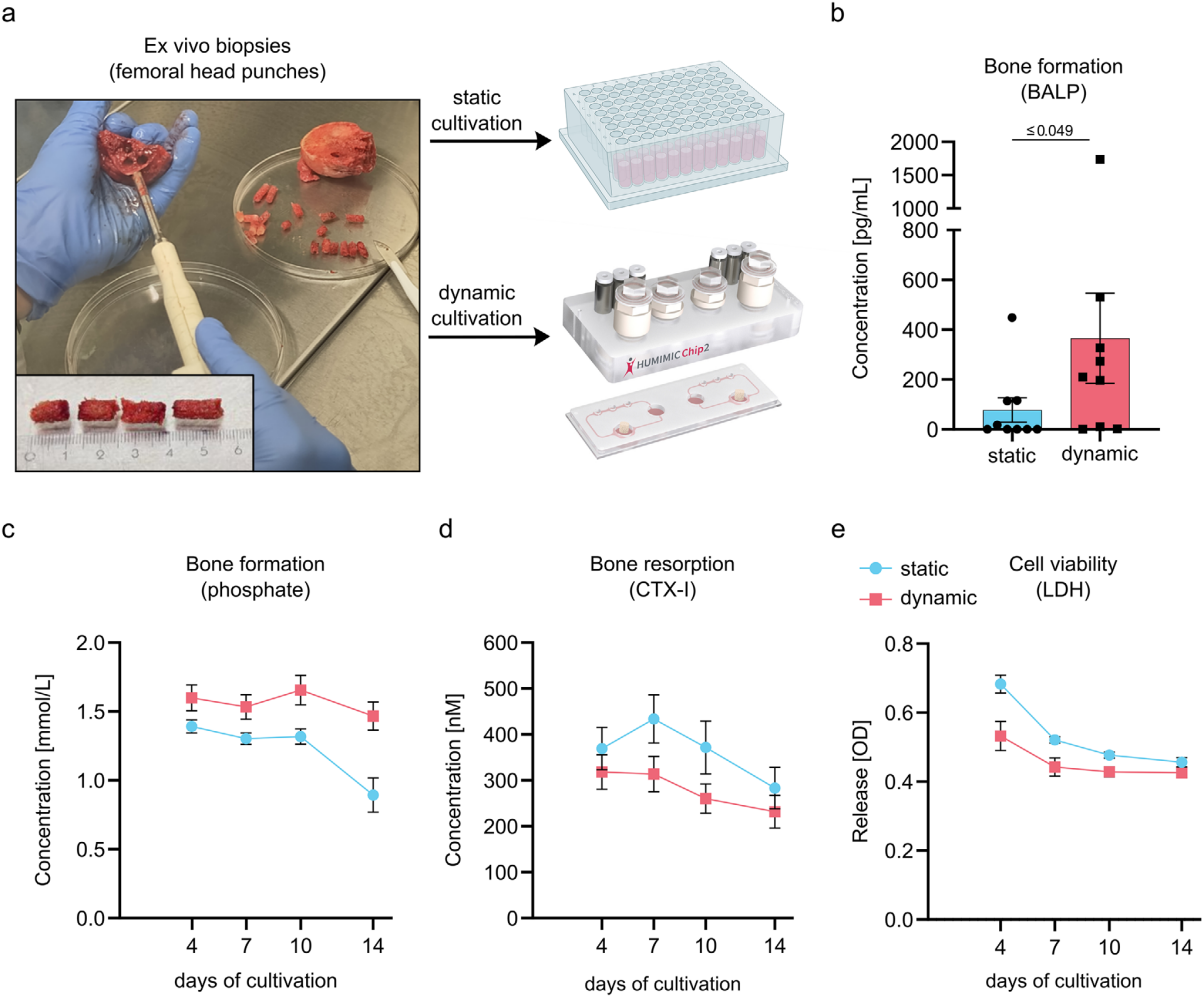
Bone tissue biopsies from human femoral heads provide a physiologically relevant platform for viability and functionality studies of bone cells. (a) Ex vivo biopsies (equal‐sized bone tissue punches) were extracted from femoral heads and cultivated for 14 days under static or dynamic conditions at 33°C. (b) BALP, a marker of osteogenic activity, was significantly increased in dynamic culture compared to static culture at day 14. (c) Phosphate levels, reflecting bone formation activity, remained stable throughout the 14 days of dynamic culture and decreased after 10 days in static culture. (d) CTX‐I, a marker for bone resorption, showed a modest decrease after 7 days in dynamic and static culture. (e) LDH concentrations were initially higher in static cultures than in dynamic cultures but converged over the course of the culture period (OD = 0.5 corresponded to 1 × 10^5^ dead cells). BALP: bone‐specific alkaline phosphatase; CTX‐I: c‐terminal telopeptide of type I collagen; LDH: lactate dehydrogenase; OD: optical density. For (b), a Mann–Whitney U test was performed. Sample sizes were as follows: for (b) n = 3‐4; for (c–e) n = 4–7. All data are presented as mean ± SEM. HUMIMIC Chip2 96‐well source: TissUse GmbH, licensed under CC BY ND 4.0.

**FIGURE 2:**
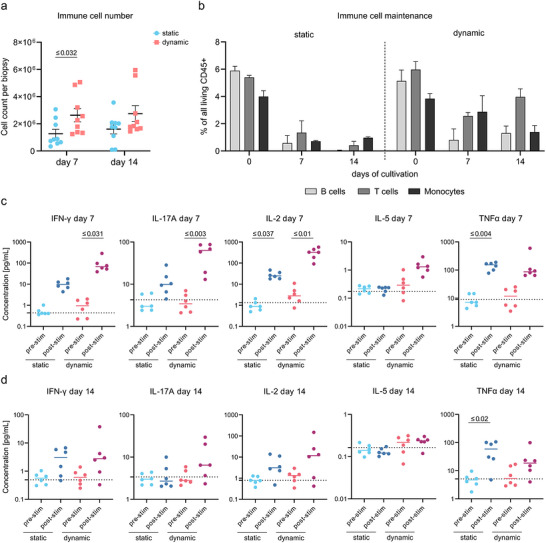
Bone tissue biopsies from human femoral heads provide a physiologically relevant platform for assessing immune cell maintenance and responsiveness. (a) Dynamic culture conditions maintained a higher number of immune cells per biopsy compared with static culture. (b) The proportion of immune cell populations declined more rapidly under static than under dynamic conditions. After 14 days, B cells were no longer detectable in static culture. (c) Con A treatment induced T cell‐associated cytokine secretion in both culture conditions on day 7, with overall higher cytokine levels in dynamic culture compared with static culture. (d) Following Con A treatment, T cell‐associated cytokine concentrations were markedly reduced after 14 days of culture compared with 7‐day cultures. Dashed lines in (c) and (d) indicate the mean concentration of unstimulated controls. Ctrl: control; IFN‐γ: interferon‐gamma; IL‐17A: interleukin‐17A; IL‐2: interleukin‐2; IL‐5: interleukin‐5; post‐stim: after stimulation; pre‐stim: before stimulation; TNF‐α: tumor necrosis factor‐alpha. For (a), (c), and (d), a Kruskal–Wallis test followed by Dunnett's multiple comparison test was used. Sample sizes were as follows: for (a), (c), (d) n = 4‐7; for (b) n = 3‐4; All data are presented as mean ± SEM.

To assess the impact of perfusion on bone homeostasis and immune cell maintenance, human femoral head biopsies of defined size were cultured under static and controlled dynamic perfusion conditions in a 3D microfluidic culture system (Figures 1 and 2). These ex vivo biopsies were cultured at 33°C for 14 days, reflecting the lower physiological temperature of femoral BM [27, 28]. Soluble bone turnover markers were monitored during the cultivation.

Markers of osteogenic activity, resorption, and viability remained stable or improved under dynamic culture conditions compared to static controls (Figure 1b–e), highlighting the beneficial effects of fluid flow on tissue function. In particular, secretion of bone‐specific alkaline phosphatase (BALP) was markedly increased in dynamic cultures, reaching 365 pg/mL at day 14 compared to 77 pg/mL in static cultures (Figure 1b). This elevation, together with stable phosphate concentrations of approximately 1.5 mmol/L over 14 days (Figure 1c) indicates enhanced osteogenic activity under dynamic conditions. Taken together, BALP activity, phosphate release, and associated matrix‐related readouts are consistent with osteogenic cellular activity in the biopsy cultures. Levels of C‐terminal telopeptide of type I collagen (CTX‐I), a marker of bone resorption, remained detectable but gradually declined over time in dynamic cultures from 320 nm on day 4 to 230 nm on day 14. In static cultures, CTX‐I levels declined from 370 to 280 nm, with a moderate transient increase from day 4 to day 7 (Figure 1d). Assessment of lactate dehydrogenase (LDH) release as a viability marker revealed a slight overall decrease, with peak levels on day 4 (OD = 0.53 in dynamic culture; OD = 0.68 in static culture), followed by stabilization in both groups by day 14 (OD ∼ 0.44; Figure 1e). These findings indicate that dynamic flow conditions support bone cell activity and tissue stability during ex vivo culture.

To evaluate the impact of dynamic cultivation on immune cell populations, non‐adherent cells were quantified, characterized and functionally assessed following cultivation (Figure 2). Dynamic culture conditions maintained a substantially higher number of immune cells, with 2.63 × 10^6^ at day 7 and 2.74 × 10^6^ at day 14, compared to static conditions, which maintained 1.27 × 10^6^ at day 7 and 1.61 × 10^6^ at day 14 (Figure 2a). In addition to preserving overall cell numbers, dynamic culture also improved the maintenance of major immune cell subsets compared with static culture. Under dynamic conditions, B cells decreased from 5.1% on day 0 to 1.3% on day 14, T cells decreased from 6 to 3.7%, and monocytes decreased from 3.8 to 1.4%. In contrast, immune cells survived at lower rates under static conditions: B cells decreased from 5.9% on day 0 to 0.05% on day 14, T cells decreased from 5.4 to 0.5%, and monocytes decreased from 4 to 1%. (Figure 2b). To assess the immune cell responsiveness during cultivation, biopsies maintained under dynamic or static conditions were treated on day 7 or day 14 for 24 h with Concanavalin A (Con A), a potent mitogen inducing T‐cell activation [29]. T cell‐ associated cytokine concentrations were quantified in the supernatant before and after stimulation and compared to unstimulated controls (Figure 2c–d). After 7 days of dynamic culture, stimulated biopsies released markedly higher levels of cytokines (101 pg/mL IFN‐γ, 55 pg/mL IL‐17A, 324 pg/mL IL‐2, 1.5 pg/mL IL‐5, 166 pg/mL TNF‐α) compared to biopsies cultured statically for 7 days (10 pg/mL IFN‐γ, 12 pg/mL IL‐17A, 28 pg/mL IL‐2, 0.2 pg/mL IL‐5, 140 pg/mL TNF‐α; Figure 2d). Following 14 days in culture, cytokine secretion upon stimulation was reduced in both conditions. Dynamic cultures reached 8 pg/mL IFN‐γ, 11 pg/mL IL‐17A, 33 pg/mL IL‐2, 0.22 pg/mL IL‐5, and 30 pg/mL TNF‐α, whereas static cultures produced 3.3 pg/mL IFN‐γ, 4.1 pg/mL IL‐17A, 5.6 pg/mL IL‐2, 0.13 pg/mL IL‐5, and 60 pg/mL TNF‐α. At this later time point, differences between dynamic and static conditions were less pronounced than those observed at day 7 (Figure 2e). Taken together, these findings indicate that dynamic cultivation is not only advantageous for bone remodeling processes but also supports the maintenance of immune cell populations and preserves their ability to respond to defined external stimulation in vitro.

### 2.2 Refined Differentiation Protocols Enable Osteogenic Matrix Deposition and Resorption in 2D Cultures

**FIGURE 3:**
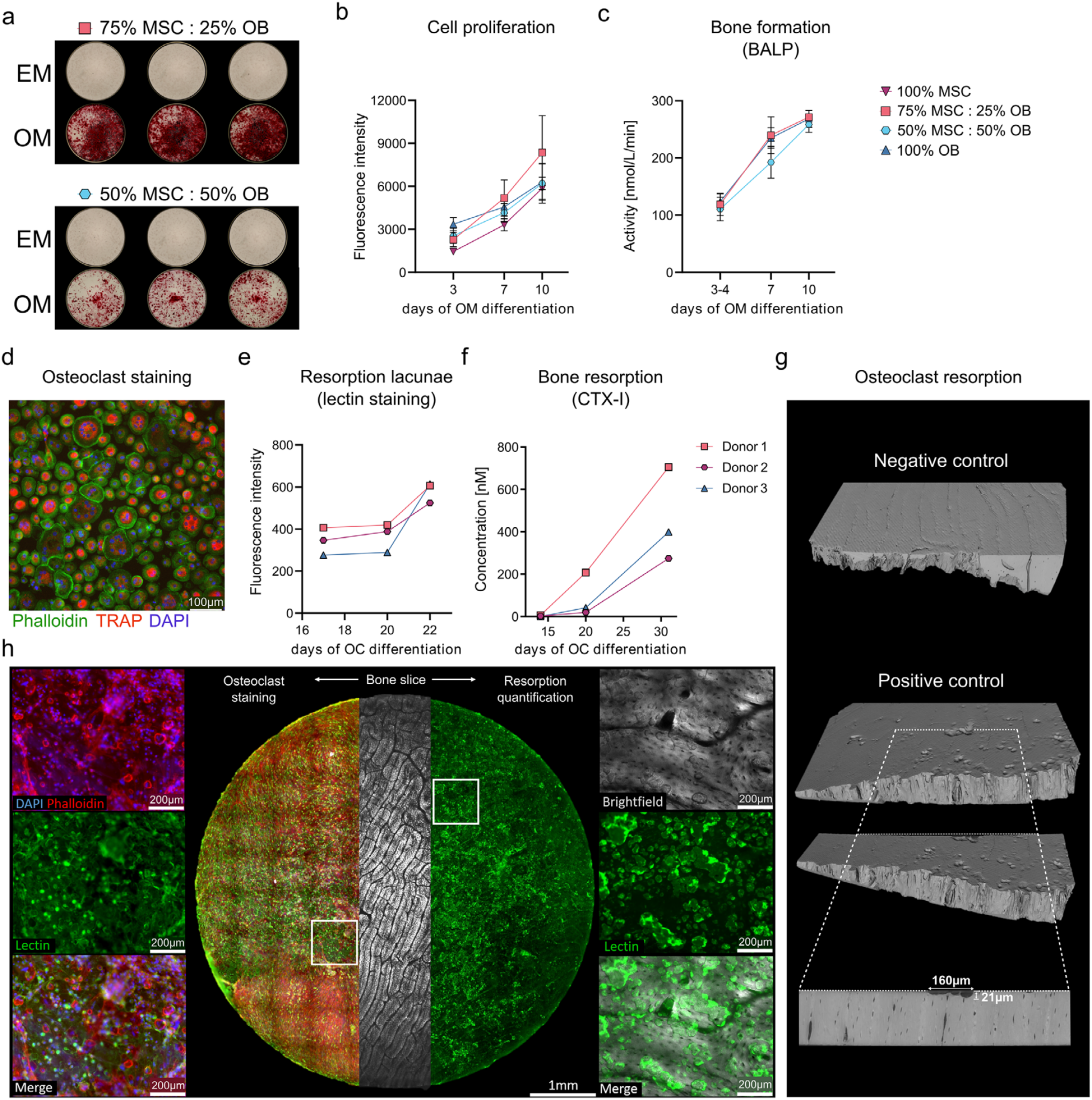
Adaptation of differentiation protocols enables osteogenic matrix formation and resorption in 2D cultures. (a) Mineralized matrix deposition was visualized by Alizarin Red staining on day 17 of osteogenic differentiation. (b) Cell proliferation was assessed by fluorescence‐based total DNA quantification. (c) BALP activity was quantified by measuring the turnover of pNPP to 4‐nitrophenolate on days 3–4, 7, and 10. (d) Multinucleated OCs with characteristic actin ring (green, phalloidin) and TRAP (red) expression were observed after 21 days of stimulation of bone marrow‐derived monocytes. (e) OC activity was quantified by lectin staining of resorption lacunae, and (f) by measuring CTX‐I release during cultivation. (g) OC‐mediated resorption was further visualized by µCT imaging. (h) OC differentiation was additionally confirmed by cell‐specific staining and quantification of resorption pits on the same bone slice. BALP: bone‐specific alkaline phosphatase; CTX‐I: c‐terminal telopeptide of type I collagen; DAPI: 4′,6‐diamidino‐2‐phenylindole; DNA: deoxyribonucleic acid; EM: expansion medium; MSC: mesenchymal stromal cell; µCT: micro‐computed tomography; OB: osteoblast; OC: osteoclast; OM: osteogenic differentiation medium; pNPP: para‐nitrophenyl phosphate; TRAP: tartrate‐resistant acid phosphatase. For (a) and (b), a two‐way ANOVA followed by Tukey's multiple comparison test was performed. Sample sizes were as follows: for (b) n = 3‐4; for (c) n = 9; for (e)‐(f) n = 3. All data presented as mean ± SEM.

Based on findings from ex vivo biopsy cultures, our goal was to generate a defined, modular BOAC platform using primary human cells. First, we refined 2D differentiation protocols to ensure robust and reproducible bone matrix formation and resorption. The production of bone matrix and the calcification process depend on the ratio of mesenchymal stromal cells (MSCs) to osteoblasts (OBs). A higher MSC‐to‐OB ratio (75% to 25%) resulted in an elevated mineralized matrix deposition (Figure 3a) and was associated with a higher proliferation rate compared to cultures with lower ratios (50% MSCs/ 50% OBs, 100% OBs, and 100% MSCs; Figure 3b). Mineralized matrix deposition was accompanied by increased BALP activity, indicating active osteogenic differentiation rather than passive mineral precipitation. BALP activity was higher in cultures with 75% MSCs to 25% OBs ratio between day 3 and day 10 compared to cultures with 50% MSCs and 50% OBs, but remained comparable to monocultures (100% OBs or MSCs; Figure 3c).

To differentiate BM‐derived monocytes into osteoclasts (OCs), we adapted a previously published protocol [30]. After 21 days, mature OCs exhibiting characteristic phalloidin‐stained ruffled membranes and tartrate‐resistant acid phosphatase (TRAP) expression were observed (Figure 3d). To evaluate OC function, bone slices were used as a substrate in resorption assays. Fluorescent lectin staining was used to quantify resorption lacunae and revealed detectable OC‐mediated bone resorption beginning on day 16 (Figure 3e). This was confirmed by measurement of OC activity via CTX‐I concentration in the culture supernatant (Figure 3f). Additionally, resorption lacunae on bone slices were visualized via micro‐computed tomography (µCT; Figure 3g), as well as by combined lectin and osteoclast marker staining to provide direct morphological evidence (Figure 3h).

### 2.3 2D Coculture Experiments Identify Optimal Seeding Order to Enable Active Osteogenic Matrix Deposition and Bone Resorption

**FIGURE 4:**
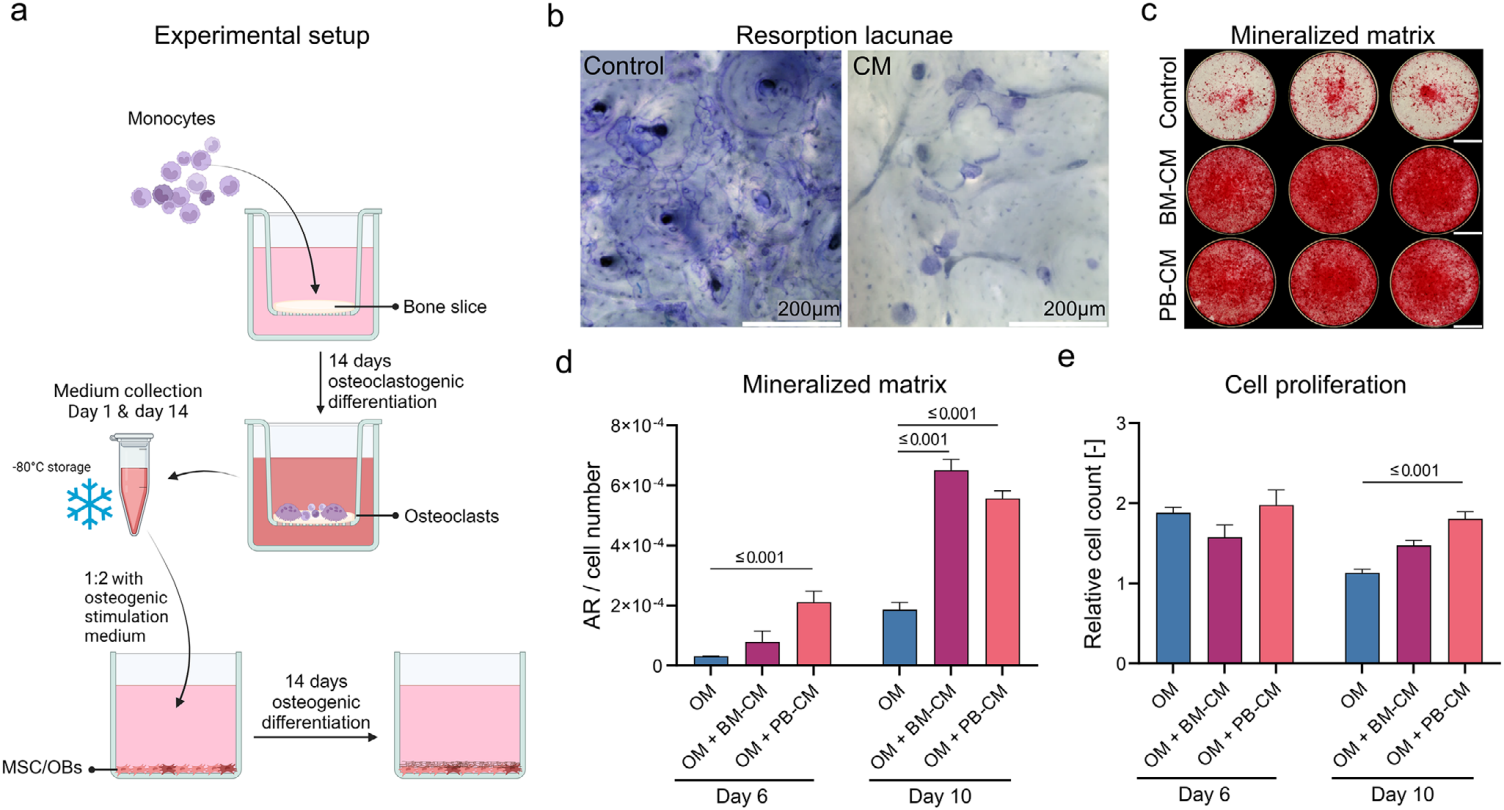
CM from MSC/OBs impairs OC function, while osteogenic differentiation is enhanced by OC‐derived CM. (a) CM was collected to study the effects of soluble factors on osteoclastogenic and osteogenic differentiation. (b) Toluidine Blue staining after 21 days of OC differentiation demonstrates impaired resorption in cultures exposed to MSC/OB‐derived CM (right) compared with the control (left). (c) AR staining of MSC/OBs at day 10 reveals enhanced matrix deposition in response to CM from BM‐ or PB‐derived OCs, relative to control (OM). Scale bar in (c): 3 mm. (d) Quantification of AR‐positive mineralized matrix deposition shows a significant increase starting from day 6 in cultures treated with OC‐derived CM. (e) Proliferation of MSC/OBs is significantly elevated by OC‐derived CM at day 10 of osteogenic differentiation. AR: Alizarin Red; BM: bone marrow; CM: conditioned medium; MSC/OBs: mesenchymal stromal cells/osteoblasts; OC: osteoclast; OM: osteogenic differentiation medium; PB: peripheral blood. For (d) and (e), a two‐way ANOVA followed by Tukey's test was used. Experiments were performed using cells from two independent donors, each analyzed in five technical replicates. Data are presented as mean ± SEM. (a) was created with BioRender.com (https://BioRender.com/5ws84g4).

The refined differentiation protocols provide a reliable basis for generating the key cell types involved in bone resorption and formation. We subsequently explored the interaction between OC and OB differentiation using indirect coculture systems based on conditioned media (CM; Figure 4a). We investigated both peripheral blood (PB)‐derived and bone marrow (BM)‐derived monocytes as sources for OCs in order to adapt the system to different applications.

For the indirect cocultures, PB‐OCs or BM‐OCs were differentiated on bone slices, and their conditioned media (PB‐CM and BM‐CM, respectively) were collected on day 1 (monocyte stage) and day 14 (macrophage/osteoclast stage). PB‐CM or BM‐CM were added to MSC/OB cultures at the beginning of osteogenic stimulation (OM). Similarly, CM from MSC/OBs was collected on day 1 (unstimulated state) and day 14 (matrix deposition stage) and subsequently added to OC differentiation cultures (Figure 4a). CM from MSC/OBs suppressed the formation of resorption lacunae during OC differentiation, whereas PB‐CM or BM‐CM promoted matrix mineralization in MSC/OBs (Figure 4b,c). Quantitative analyses revealed that MSC/OBs exposed to OC‐derived CM exhibited an approximately threefold increase in mineralized matrix deposition by day 10 relative to controls (Figure 4d). Proliferation of MSC/OBs was only marginally enhanced, primarily by PB‐CM (Figure 4e). These findings demonstrate a reciprocal regulatory relationship between OCs and MSC/OBs through soluble factors, highlighting the importance of cell–cell communication in bone homeostasis.

**FIGURE 5:**
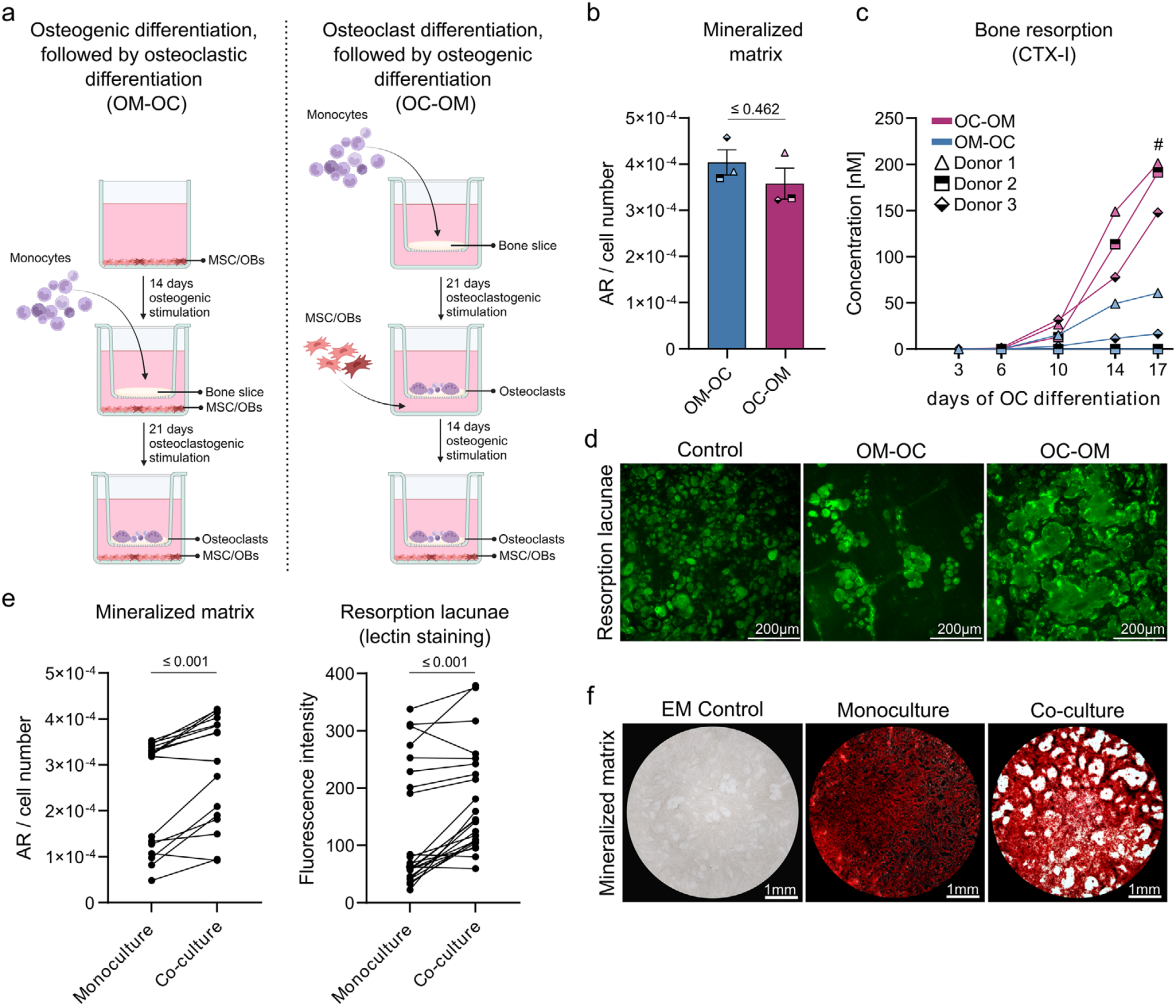
Direct 2D cocultures reveal that OCs are impacted by soluble factors from MSC/OBs, while osteogenic differentiation remains unaffected. (a) Two sequential coculture strategies were tested using BM‐derived OCs and autologous MSC/OBs: osteogenic cells were seeded and differentiated first, followed by OCs (OM‐OC), or OCs were seeded and differentiated prior to osteogenic cells (OC‐OM). (b) Mineralized matrix quantification, normalized to MSC/OB cell number, showed that osteogenic differentiation was unaffected by the seeding sequence. (c) CTX‐I levels in culture supernatant indicated reduced osteoclast activity in OM‐OC compared with OC‐OM cocultures. (d) Lectin‐stained bone slices revealed fewer and smaller resorption lacunae in OM‐OC cultures, while OC‐OM cultures showed larger resorption areas than OC monocultures. (e) Both matrix mineralization and resorption were enhanced in OC‐OM coculture compared with the respective monocultures. (f) Alizarin Red staining of MSC/OB compartment showed morphological changes in the cell layer between monoculture and coculture after 14 days of osteogenic differentiation. No AR‐positive matrix deposition was detected in the coculture negative control (EM, expansion medium without osteogenic stimulus). AR: Alizarin Red; BM: bone marrow; CTX‐I: c‐terminal telopeptide of type I collagen; EM: expansion medium; MSC/OBs: mesenchymal stromal cells/osteoblasts; OC: osteoclast. For (b), (c), and (e), paired t‐tests were used. Sample sizes were as follows: for (b) and (c) n = 3; for (e) n = 6–8. Results are presented as mean ± SEM. “#” indicates p = 0.014 between OC‐OM and OM‐OC on day 17. (a) was created with BioRender.com (https://BioRender.com/187tkzv).

To examine direct interactions between OCs and MSC/OBs, we used a second 2D coculture approach. MSC/OBs were differentiated at the bottom of a 24‐well plate, while monocytes underwent OC differentiation on bone slices in a cell culture insert. Both compartments shared the same medium. Two differentiation sequences were tested: In the OM‐OC group, the MSC/OBs were first osteogenically differentiated for 14 days, and then the monocytes were introduced for OC differentiation. In the OC‐OM group, the monocytes were differentiated into OCs for 14 days prior to the addition of the MSC/OBs, which were then differentiated osteogenically (Figure 5a). MSC/OB mineralized matrix deposition was not influenced by the differentiation sequence, as OM‐OC and OC‐OM cultures exhibited similar levels of Alizarin Red‐positive matrix (Figure 5b). In contrast, OC activity was highly dependent on the differentiation sequence. The OM‐OC group displayed significantly fewer resorption lacunae and a lower CTX‐I concentration than the OC‐OM group (CTX‐I: OM‐OC = 50 nm; OC‐OM = 175 nm, p = 0.014; Figure 5c,d). To validate these results, we compared the OC‐OM differentiation strategy with monocultures of similar duration. This analysis showed that the OC‐OM seeding sequence enhanced both matrix mineralization and resorption compared to the corresponding monocultures (Figure 5e). Direct cocultures resulted in the formation of gaps (similar to bone nodules) within the previously confluent MSC/OB cell layer, a phenomenon not observed in monoculture (Figure 5f). These findings demonstrate that the seeding order is an important parameter for generating functional bone remodelling in vitro. Sequential seeding starting with OC differentiation on the bone scaffold is crucial for preserving the functional activity of both bone cell types.

### 2.4 The Cultivation Temperature Critically Influences In Vitro Bone Resorption and Osteogenic Activity

Although the temperature in peripheral BM is lower than in the core body [27, 31, 32], the optimal temperature for simultaneously supporting bone formation and resorption compartments in vitro remains unclear. Previous studies have demonstrated that 33°C is superior to 37°C for the long‐term in vitro maintenance of immune cells and hematopoiesis [28, 33], whereas 37°C promotes accelerated growth of the stromal layer [28]. Therefore, we investigated the impact of 33°C and 37°C on OB and OC activity.

**FIGURE 6:**
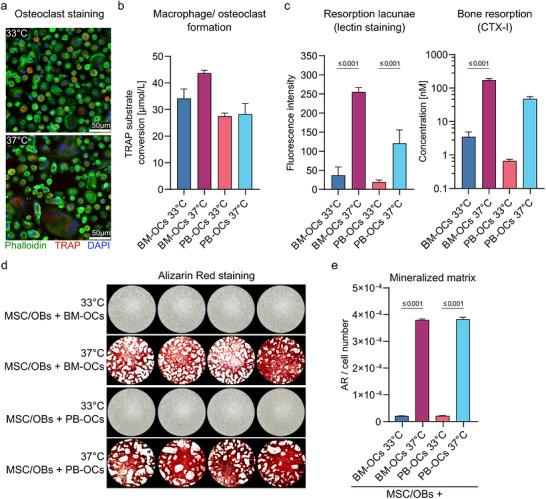
Bone cell differentiation is accelerated at 37°C compared with 33°C. (a) Immunofluorescence staining of BM‐OCs after 14 days of differentiation shows increased cell size at 37°C compared with 33°C. Representative images show nuclei (DAPI, blue), phalloidin (green), and TRAP (red). (b) TRAP activity, a marker for macrophages and osteoclasts, remained comparable across temperatures in both BM‐OCs and PB‐OCs. (c) Osteoclast function, assessed by resorption pit quantification and CTX‐I levels in the supernatant, was elevated at 37°C compared with 33°C. (d) AR staining reveals mineralized matrix formation in cocultures at 37°C, while no matrix was observed at 33°C after 14 days of osteogenic differentiation. (e) Quantification of AR‐positive matrix deposition, normalized to cell number (Hoechst staining), demonstrates enhanced osteogenic differentiation at 37°C after 28 days. AR: Alizarin Red; BM‐OCs: bone marrow‐derived osteoclasts; CTX‐I: c‐terminal telopeptide of type I collagen; DAPI: 4′,6‐diamidino‐2‐phenylindole; MSC/OBs: mesenchymal stromal cells/osteoblasts; PB‐OCs: peripheral blood‐derived osteoclasts; TRAP: tartrate‐resistant acid phosphatase. For (b), (c), and (e), a one‐way ANOVA with Tukey's multiple comparison test was performed; n = 2–3 biological replicates per group. Results are presented as mean ± SEM.

Functional experiments in direct coculture systems revealed that OCs exhibited increased size and activity at 37°C compared to 33°C. Although TRAP activity was comparable between conditions and depended only on the origin of the monocytes (BM versus PB), 37°C led to higher resorption pit numbers. Consequently, higher CTX‐I concentrations were measured in 37°C cultures compared to the 33°C conditions (Figure 6a–c). Mineralized matrix formation was exclusively observed at 37°C (Figure 6d,e). These results established 37°C as the optimal temperature for bone cell differentiation.

### 2.5 Dynamic Flow Promotes Immune Cell Maintenance and Bone Remodeling Markers in 3D BOAC Monocultures

**FIGURE 7:**
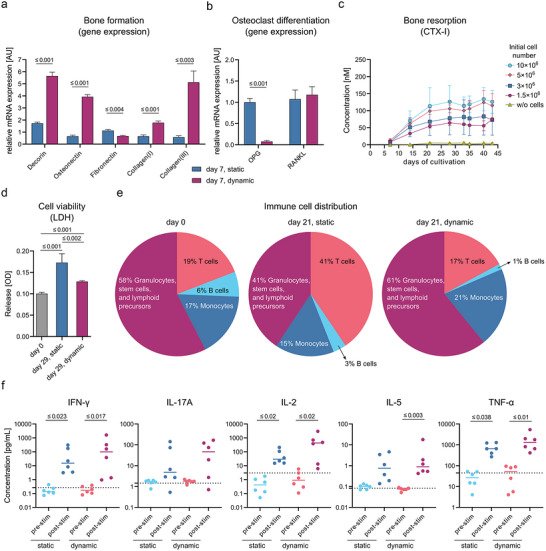
Dynamic medium flow supports BM homeostasis and function, matrix‐associated gene expression, and OC activity in 3D cultures. (a) Dynamic culture showed increased expression of osteonectin, decorin, collagen (I), and collagen (III), while the expression of fibronectin was reduced. (b) In static culture, both OPG and RANKL were expressed, whereas dynamic culture conditions resulted in comparable RANKL but minimal OPG expression. (c) CTX‐I concentration increased and stabilized in dynamic long‐term monocultures (42 days), depending on the initial number of PB‐monocytes. (d) Cytotoxicity was significantly lower under dynamic conditions compared with static culture after 29 days. (e) Static culture resulted in a shift among the major immune cell subsets. In a dynamic culture, cell subset distribution remained closer to baseline (day 0), with a slight increase in monocytes and a decrease in T and B cells. (f) Con A treatment at day 14 induced T cell‐associated cytokine secretion under both static and dynamic conditions, with higher concentrations observed in dynamic culture. For (a) values were normalized to day 0 (d0) in the expansion medium. For (b) values were normalized to the static condition. Dashed lines in (f) indicate the mean concentration of unstimulated controls. CTX‐I: c‐terminal telopeptide of type I collagen; IFN‐γ: interferon‐gamma; IL‐17A: interleukin‐17A; IL‐2: interleukin‐2; IL‐5: interleukin‐5; LDH: lactate dehydrogenase; mRNA: messenger ribonucleic acid; OPG: osteoprotegerin; PB: peripheral blood; RANKL: receptor activator of nuclear factor‐κB ligand; TNF‐α: tumor necrosis factor‐alpha. For (a) and (b), an unpaired t‐test was performed; for (d), one‐way ANOVA followed by Tukey's multiple comparison test; and for (f), a Kruskal‐Wallis test followed by Dunnett multiple comparison test was performed. Sample sizes were as follows: for (a), (b), and (d) n = 4; for (c) and (f) n = 2‐3; for (e) n = 7–10. Results are presented as mean ± SEM.

We tested the refined differentiation protocols in static and dynamic 3D monocultures of MSC/OBs or monocytes, as well as PB/BM‐mononuclear cells (PBMCs/BMMNCs) using decellularized bone scaffolds. Gene expression of markers associated with ECM formation, such as osteonectin, decorin, collagen type I, and collagen type III, was significantly higher in dynamic than in static MSC/OB cultures (Figure 7a). Fibronectin levels decreased under dynamic conditions. Osteoprotegerin (OPG) expression was absent in dynamic MSC/OB monocultures, whereas expression of receptor activator of nuclear factor‐κB ligand (RANKL) was unaffected by dynamic medium flow (Figure 7b). OC differentiation and active resorption in dynamic monocyte cultures were monitored via CTX‐I release.

Osteoclastic activity increased over time and correlated with the number of monocytes used to colonize the 3D bone scaffold, indicating successful osteoclastogenesis and maintenance for up to 42 days (Figure 7c). LDH cytotoxicity assays indicated an increase in LDH levels under both conditions after 29 days, with significantly higher LDH release under static conditions, indicating elevated cell death (Figure 7d). Static 3D BMMNC monocultures exhibited pronounced shifts in cellular composition during cultivation (Figure 7e). On day 0 (baseline), the cultures consisted of 19% T cells, 6% B cells, 17% monocytes, and 58% other cells. By day 21, this composition had shifted to 41% T cells, 3% B cells, 15% monocytes, and 41% other cells under static conditions. In contrast, dynamic culture conditions maintained 17% T cells, 1% B cells, 21% monocytes, and 61% other cells at day 21, thereby more closely reflecting the baseline composition. Immune cell responsiveness to defined stimulation was assessed by treating PBMC monocultures with Concanavalin A (Con A) after 14 days of prior cultivation under static and dynamic conditions. Both culture conditions showed an increase in the concentration of T cell‐associated cytokines compared to pre‐stimulation levels and unstimulated controls (dashed line). Overall, cytokine concentrations were higher in dynamic cultures, with 354 pg/mL IFN‐γ, 64 pg/mL IL‐17A, 760 pg/mL IL‐2, 3.7 pg/mL IL‐5, and 1 825 pg/mL TNF‐α, compared to static cultures, which showed 86 pg/mL IFN‐γ, 38 pg/mL IL‐17A, 67 pg/mL IL‐2, 1.6 pg/mL IL‐5, and 745 pg/mL TNF‐α (Figure 7e). These data suggest that dynamic culture conditions improve cell survival, stabilize immune cell composition, and preserve the ability of immune cells to respond to defined stimulation in the 3D bone environment.

### 2.6 BOAC Model Integrates Active Resorption, Osteogenic Matrix Formation, and Immune Cell Maintenance Under Dynamic Conditions

**FIGURE 8:**
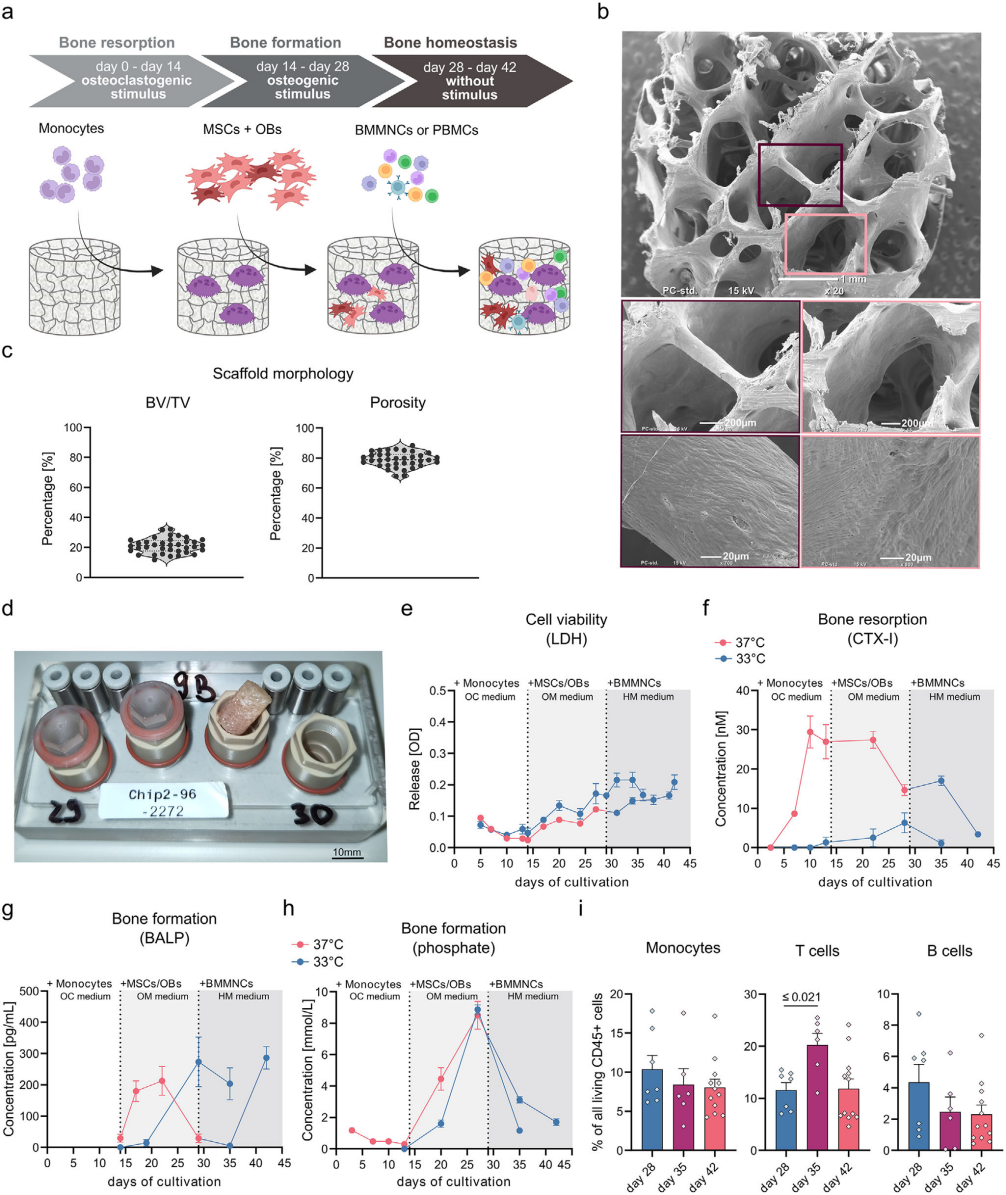
Elevated temperature is required for functional 3D bone remodeling, while lower temperature supports immune cell homeostasis. (a) Colonization of the native bone scaffold was performed in three phases: First, monocytes were differentiated into OCs over 14 days. Second, MSCs and OBs were added and cocultured under osteogenic conditions for an additional 14 days. In the final phase, mononuclear immune cells were introduced and maintained without further stimulation for an additional 14 days. (b) Representative SEM images of the decellularized bone scaffold were acquired to assess surface structural characteristics prior to colonization. (c) Morphological parameters of each scaffold were determined by µCT scanning and analysis prior to colonization (see Table S1 for complete data). (d) After 5 weeks of culture, soft tissue‐like structures developed between the trabeculae, and a vertical red‐to‐yellow gradient becomes visible within the scaffold. (e) LDH concentration increased over time but remained below the cytotoxic threshold (OD = 0.5, equivalent to 1×10^5^ dead cells). (f) CTX‐I concentrations, reflecting OC activity, increased in the 37°C culture compared with the 33°C culture and remained elevated over the cultivation period. (g) BALP concentration increased in both cultures during osteogenic stimulation and reached its peak sooner in the 37°C culture. (h) Phosphate concentration in the supernatant increased similarly in both cultures during osteogenic differentiation. (i) Flow cytometry analysis of BMMNC populations in primary BOAC cultures revealed immune cell maintenance over 14 days under dynamic conditions. BALP: bone‐specific alkaline phosphatase; BMMNCs: bone marrow mononuclear cells; BOAC: Bone‐on‐a‐Chip; BV: bone volume; CTX‐I: c‐terminal telopeptide of type I collagen; FACS: fluorescence‐activated cell sorting; HM medium: homeostasis medium; LDH: lactate dehydrogenase; MSC/OBs: mesenchymal stromal cells/osteoblasts; OCs: osteoclasts; OC medium: osteoclast differentiation medium; OD: optical density; OM medium: osteogenic differentiation medium; PBMCs: peripheral blood mononuclear cells; TV: total volume. For (i), a one‐way ANOVA followed by Dunnett's multiple comparison test was performed. Sample sizes were as follows: for (c) n = 36; for (e‐h) n = 6–16; and for (i) n = 6‐12. Results are presented as mean ± SEM. (a) was created with BioRender.com (https://BioRender.com/so2xzsm).

To further validate the findings from 2D coculture assays in the 3D environment, we sequentially seeded BM‐derived monocytes, MSC/OBs, and BMMNCs onto decellularized human cancellous bone scaffolds to generate a complex, multicellular BOAC model (Figure 8a). Surface structures of these bone scaffolds were assessed using scanning electron microscopy (SEM; Figure 8b). Morphological parameters of each scaffold prior to cultivation were obtained by µCT scanning and analysis. The decellularized bone scaffolds exhibited a mean BV/TV of 21.1 ± 0.8%, a porosity of 78.9 ± 0.8%, a trabecular thickness of 166.6 ± 4.7 µm, and a mean pore diameter of 726.5 ± 22.8 µm, indicating low variability across scaffolds (Figure 8c and Table S1). These values fall within the reported range for human trabecular bone microarchitecture, where BV/TV typically ranges between 12–30%, trabecular thickness between ∼110–250 µm, and porosity between 70–90% (please refer to Table S2). Based on the average fluid flow rate (6.3 ± 0.8 µL/min) and scaffold geometry, the superficial flow velocity across the scaffold was estimated at ∼3.7 µm/s. Accounting for the mean scaffold porosity, this corresponds to an interstitial pore velocity of ∼4.7 µm/s. Using the mean pore diameter determined by µCT, the resulting shear stress was estimated to be approximately in the µPa range for culture medium (∼6 µPa) and in the sub‐Pa range (∼0.5 Pa) when assuming marrow‐like effective viscosity [34].

For the establishment of the BOAC cultures, monocytes were first seeded onto the scaffolds and differentiated into osteoclasts over 14 days. This was followed by the addition of MSC/OBs, which were subsequently induced to differentiate osteogenically for another 14 days. BMMNCs were introduced, and the system was maintained for either 7 or 14 days without any differentiation stimulus (Figure 8a). Two distinct temperature conditions were evaluated: one in which all cell types were cultured at 33°C (designated as the 33°C condition) and another in which OCs and MSC/OBs were maintained at 37°C, while BMMNCs remained at 33°C (designated as the 37°C condition). After five weeks of dynamic cultivation at 33°C, scaffold trabeculae showed soft tissue‐like structure formation and a “subtle vertical color gradient” (Figure 8d). LDH measurements indicated low cell death rates under both conditions. Osteogenic markers such as BALP and phosphate peaked earlier and at higher levels in the 37°C cultures (Figure 8e,g,h). OC activity, measured by CTX‐I concentration, was significantly increased at 37°C, with peak levels of 30 nm compared to 6 nm at 33°C (Figure 8f). FACS analysis of immune cells revealed a stable monocyte population, a transient increase in T cells during the first week that returned to baseline during the second week, and a slight reduction in B cells after one week of coculture at 33°C (Figure 8i). These results indicate that 37°C supports bone cell differentiation in 3D and is associated with increased resorption and osteogenic matrix formation, while 33°C is more suitable for maintenance of immune cell populations. Sequential differentiation of OCs and MSC/OBs was performed at 37°C in all subsequent experiments. After the final colonization of the 3D scaffolds with BMMNCs, the temperature was lowered to 33°C to support immune cell maintenance.

### 2.7 Reticular Tissue Forms Early in BOAC Cultures, While Soft Tissue Matures in Long‐Term Cultures

**FIGURE 9:**
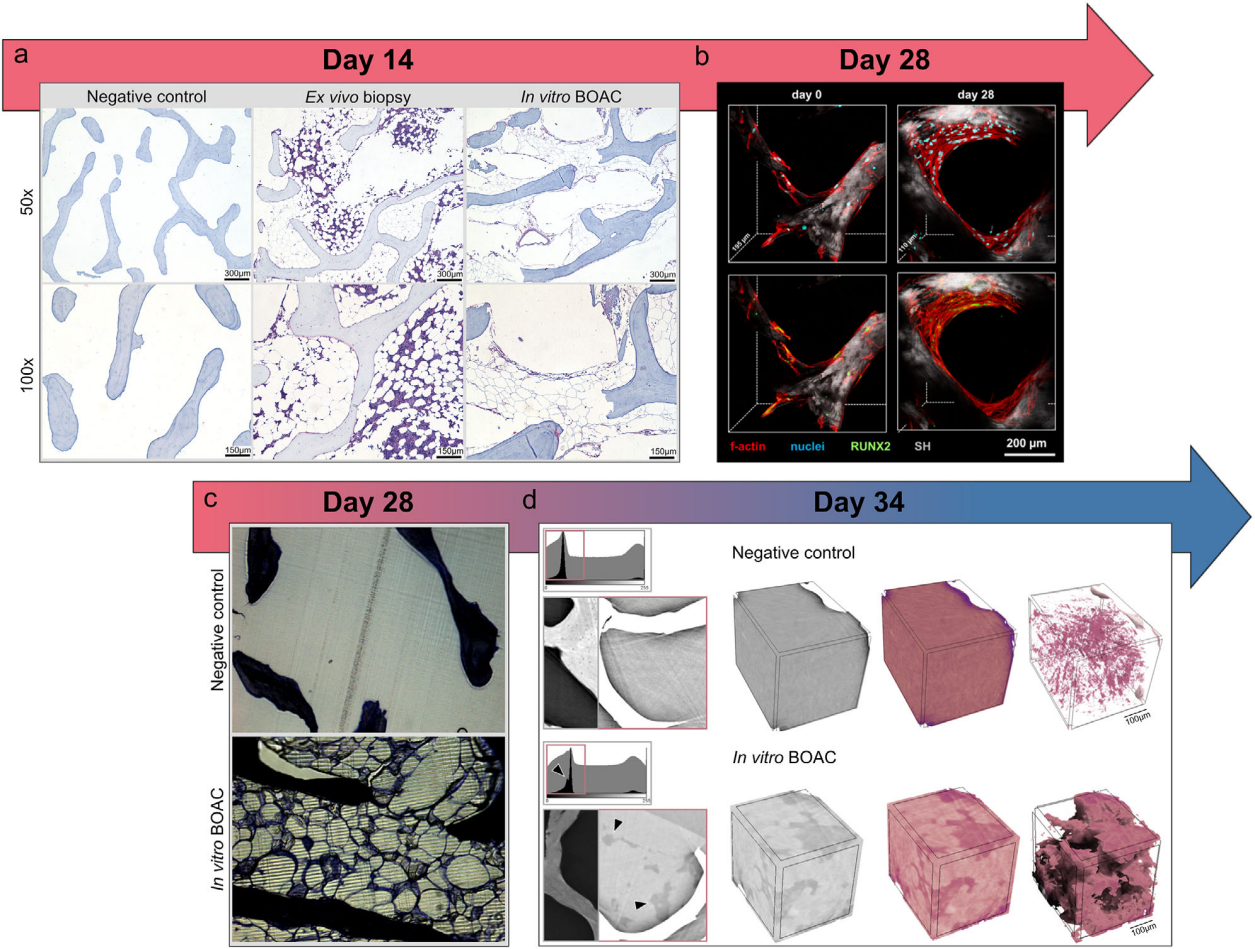
Reticular tissue structures emerge by day 14, followed by soft tissue‐like structure formation from day 34 during BOAC cultivation. (a) Hematoxylin (violet) and Cathepsin K (red) staining revealed the initial formation of reticular tissue between bone trabeculae in BOAC cultures. In the ex vivo biopsy, these regions were densely populated with BM‐derived cells. (b) Second harmonic generation imaging at day 28 demonstrated stromal cell organization, with F‐actin (red), nuclei (DAPI, blue), and RUNX2 (green), a key transcription factor for osteoblast differentiation. (c) The reticular connective tissue became increasingly structured and robust by day 28. (d) In the long‐term BOAC culture, soft tissue‐like structure became visible by synchrotron‐based µCT imaging by day 34. BOAC: Bone‐on‐a‐Chip; DAPI: 4′,6‐diamidino‐2‐phenylindole; RUNX2: Runt‐related transcription factor 2; µCT: micro‐computed tomography.

Structural organization of connective tissue within the BM cavity is essential for functional BM. Hematoxylin staining revealed early development of reticular tissue in the BOAC cultures between the bone trabeculae of colonized scaffolds by day 14 (Figure 9a). These spaces were completely filled with ECM and BM‐derived cells in the ex vivo biopsy. Stromal cell organization in the BOAC cultures became visible by day 28. Second harmonic imaging highlighted specific cytoskeletal arrangement with f‐actin and nuclear Runt‐related transcription factor 2 (RUNX2) expression (Figure 9b). At this stage, the reticular connective tissue exhibited further development and increased robustness (Figure 9c). By day 34, synchrotron‐based µCT imaging revealed further expansion of soft tissue‐like structures in the BOAC cultures, indicating a later stage of tissue organization and maturation within the system (Figure 9d). These findings underscore the sequential progression of tissue development in the BOAC cultures, from the initial reticular framework to more complex soft tissue‐like structures.

### 2.8 The Soluble Environment and Cellular Composition of BOAC Cultures Reveal Similarities to Ex Vivo Biopsies

**FIGURE 10:**
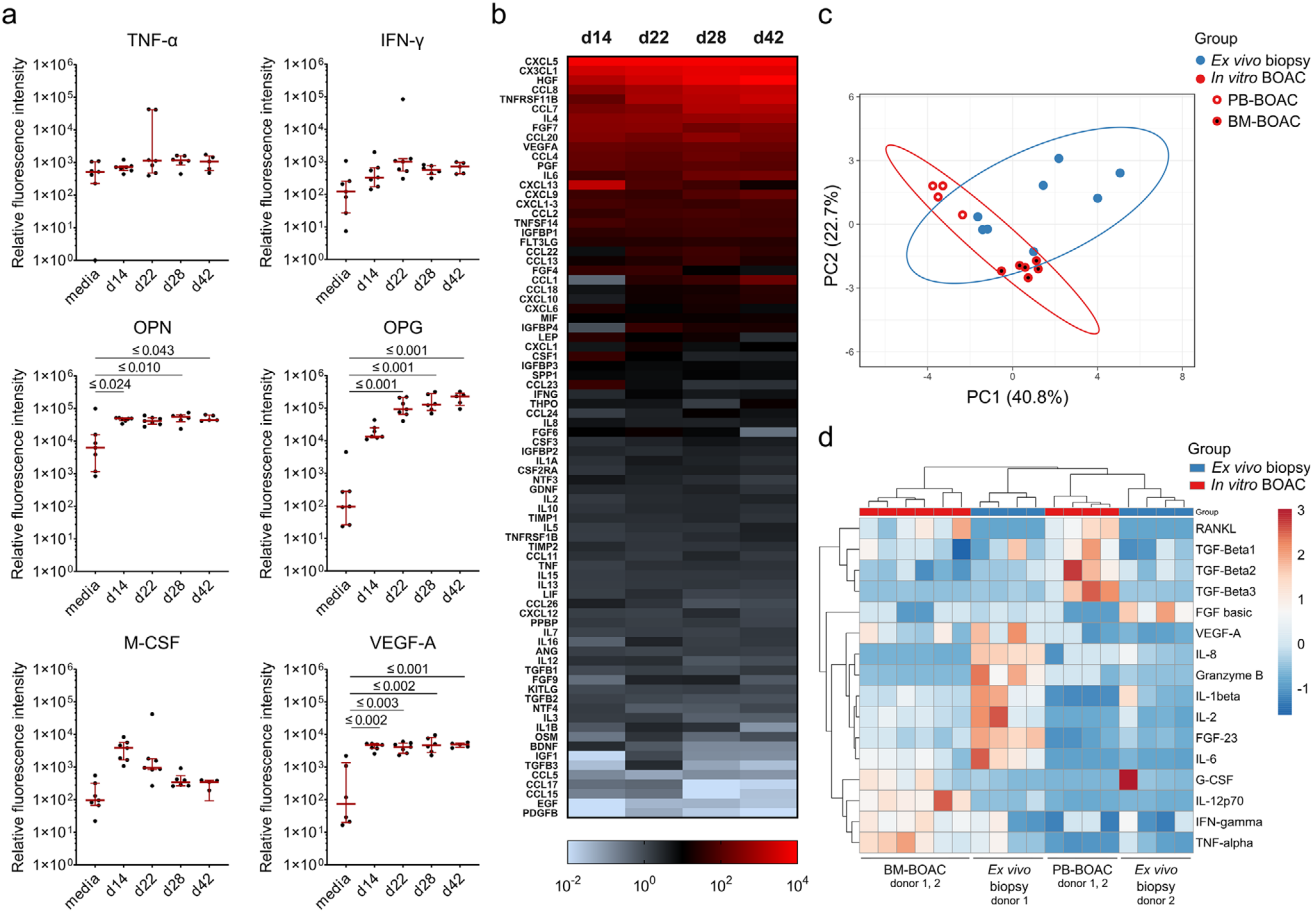
Cytokine profiling revealed a non‐inflammatory soluble environment in BOAC cultures with similarities to ex vivo bone biopsies. (a) Concentrations of pro‐inflammatory cytokines TNF‐α and IFN‐γ remained stable during long‐term BOAC culture. In contrast, osteogenic markers OPN and OPG increased over time. M‐CSF decreased, while VEGF‐A levels consistently rose from day 14 onwards. (b) Heatmap analysis showed stable profiles of soluble peptides, proteins, and cytokines throughout the cultivation period. (c) PCA analysis of 16 soluble factors at day 42 demonstrated overlap between BOAC cultures and donor‐matched ex vivo biopsies. (d) PB‐BOAC cultures displayed a closer similarity to matched biopsies than BM‐BOAC cultures. BM‐BOAC: model based on BMMNCs, IFN‐γ: interferon‐gamma, M‐CSF: macrophage colony‐stimulating factor, OPG: osteoprotegerin, OPN: osteopontin, PB‐BOAC: model based on PBMCs, PCA: principal component analysis, TNF‐α: tumor necrosis factor‐alpha, VEGF‐A: vascular endothelial growth factor A. For (a), a two‐way ANOVA followed by Dunnett's multiple comparison test was performed. Sample sizes were as follows: for (a) and (b) n = 5–7; for (c) and (d) n = 8–10. Results are presented as mean ± SEM.

Soluble factors in the cell culture supernatant play a crucial role in regulating cell behavior, interactions, and communication. These factors mediate signaling pathways that influence processes such as differentiation, proliferation, and tissue organization. In long‐term BOAC cultures, levels of pro‐inflammatory cytokines such as tumor necrosis factor‐alpha (TNF‐α) and interferon‐gamma (IFN‐γ) in the supernatant did not show a significant increase (Figure 10a). Osteopontin (OPN), a marker associated with bone formation, increased from day 14 onwards. OPG, an osteoclast regulator secreted by OBs, exhibited a consistent rise over the culture period. Macrophage colony‐stimulating factor (M‐CSF), also secreted by OBs and important for macrophage and OC differentiation, decreased over time in culture. Vascular endothelial growth factor A (VEGF‐A), a key angiogenesis factor, increased starting at day 14. The heatmap analysis, depicting all factors analyzed, revealed a constant secretion of soluble peptides, proteins, and cytokines (Figure 10b). Principle component analysis (PCA) revealed an overlap between the soluble environment of the BOAC cultures at day 42 and corresponding donor‐matched ex vivo biopsies (Figure 10c). The soluble environment depended on whether BMMNCs (BM‐BOAC) or PBMCs (PB‐BOAC) were used to generate the immune cell compartment in the final colonization step. BM‐BOAC cultures showed a greater divergence from the ex vivo biopsies than PB‐BOAC cultures. A more detailed analysis also revealed greater differences between ex vivo biopsies from distinct donors than between ex vivo biopsies and PB‐BOAC cultures from the same donor (Figure 10d).

**FIGURE 11:**
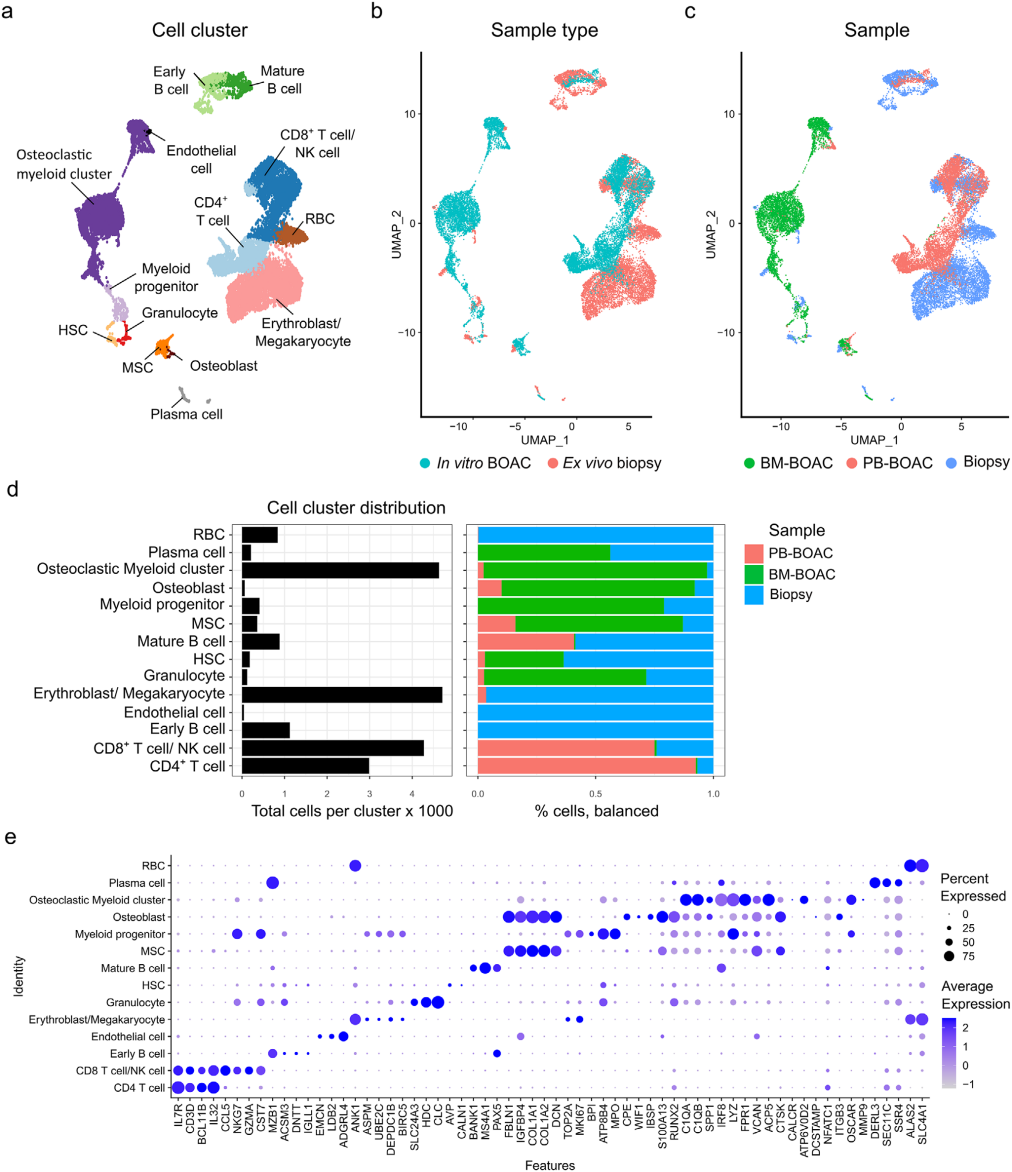
Single‐nucleus transcriptomic profiling revealed partial overlap in cellular populations between 6‐week BOAC cultures and donor‐matched ex vivo biopsy. (a) Single‐nuclei RNA sequencing identified fourteen distinct cell type clusters across two BOAC samples and one donor‐matched ex vivo biopsy. (b) Color‐coded sample mapping showed broad overlap in cell clusters between the BOAC cultures and the biopsy. (c) Detailed analysis of cell cluster distribution showed that innate immune cells were primarily retained in the BM‐BOAC (green), while adaptive immune cells were more prominent in the PB‐BOAC (red). (d) Quantitative comparison of cell cluster abundance indicated that RBCs, endothelial cells, and early B cells were only present in the biopsy and absent in both BOAC samples. (e) Dot plot showing scaled expression of cluster‐specific marker genes of the identified cell clusters. BM‐BOAC: model based on BMMNCs, BOAC: Bone‐on‐a‐Chip; HSC: hematopoietic stem cell; MSC: mesenchymal stromal cell; PB‐BOAC: model based on PBMCs; RBC: red blood cell. For (a‐d), n = 1–2 samples per group (donor‐matched datasets).

To investigate this further, the cellular composition of PB‐BOAC and BM‐BOAC cultures was analyzed after six weeks of cultivation using single‐nuclei RNA (snRNA) sequencing. The results were then compared to a donor‐matched ex vivo biopsy to explore cellular similarities and differences between the in vitro system and native bone marrow. Unsupervised clustering identified fourteen distinct cell type clusters based on nuclear RNA expression, including OB‐like cells, OC‐like cells (osteoclastic myeloid cluster) and lymphoid cells (e.g. T cells and B cells; Figure 11a). The osteoclastic myeloid cluster comprised myeloid cells that expressed markers such as CD11b, CD14, and CSF1R, indicating a monocyte/macrophage origin. It was also characterized by variable co‐expression of osteoclast‐specific genes, including *ACP5* (encoding TRAP), *CTSK*, *DCSTAMP*, *NFATC1*, *ATP6V0D2*, and *MMP9*. This suggests that the cluster contains OC precursors, intermediate differentiation stages, and functionally active resorbing cells, which differ from classical myeloid clusters by their OC‐specific transcriptional signature. Sample assignments revealed a broad overlap in cell types between the ex vivo biopsy and the BOAC cultures (Figure 11b). Innate immune cell populations, such as myeloid progenitors and cells of the osteoclastic myeloid cluster, were predominantly preserved in the BM‐BOAC. In contrast, adaptive immune cells, including T cells and B cells, were more abundant in the PB‐BOAC system (Figure 11c).

Quantitative analysis of cell clusters identified cells of the osteoclastic myeloid lineage, erythroblasts, CD8^+^ T cells, and CD4^+^ T cells as the most abundant cell types. Notably, red blood cells, endothelial cells, and early B cells were present in the ex vivo biopsy but absent in both BOAC cultures (Figure 11d). Dot plot analysis demonstrated the scaled expression of cluster‐specific marker genes expression across identified cell clusters (Figure 11e). To further contextualize the 10X Flex fixed snRNA‐seq data, publicly available human bone marrow 10×3’ single‐cell RNA‐seq datasets were incorporated as reference (Figure S1), due to the limited availability of comparable single‐nucleus datasets. Integrated analysis revealed a qualitative overlap in major immune cell populations (e.g., lymphoid and myeloid lineages) between BOAC‐derived data and public reference datasets, while bone‐associated cell populations were less consistently represented. These comparisons provide supportive context for the identified cell types in the BOAC system, but are not intended for quantitative assessment due to differences in sequencing modality, sample processing, and dataset composition. In summary, these qualitative analyses suggest that our BOAC system recapitulates selected features of human BM physiology in vitro. This includes active bone remodeling, maintenance of adult immune and stromal cell subsets, and stepwise development of reticular and soft tissue within a native bone scaffold. Histological, proteomic, and transcriptomic analyses collectively support ECM‐rich tissue organization and the emergence of a donor‐specific multicellular architecture. Single‐nucleus RNA sequencing revealed a broad overlap in major immune and bone‐related cell types compared to ex vivo BM, although certain populations, such as early B cells, endothelial cells, and erythrocytes, were not detected. However, as the snRNA‐seq analysis was performed on single replicates of each BOAC condition and the donor‐matched biopsy, the dataset is interpreted primarily as a qualitative comparison of cellular diversity rather than a quantitative assessment of population frequencies or donor variability.

## 3 Discussion

The limited cross‐species relevance of animal testing for human biology and pathology, as well as ethical issues related to the 3Rs principles, underscore the need for alternative strategies. Organ‐on‐a‐Chip platforms are a promising in vitro approach that aims to replicate the essential aspects of the complex physiology of human organs [35, 36]. Despite progress in this area, most existing bone‐related in vitro systems are based on synthetic scaffolds, animal cells, and lack the integration of adult immune cells. In addition, they are narrowly focused on either hematopoiesis or osteogenesis [37]. Our human BOAC model addresses these limitations by using a native human cancellous bone scaffold and matched autologous adult primary cells. In contrast to engineered biomaterials, the decellularized human bone scaffolds retain the structural characteristics of trabecular bone. Accordingly, scaffold characterization, including µCT‐based morphometry and SEM imaging, confirmed their physiological architecture. Previous studies have shown that the decellularization process preserves the native mineral phase of the bone, with hydroxyapatite remaining the dominant component and no significant alterations in crystallinity or composition compared to their native counterpart tissue, even across donor age groups [38].

By employing xeno‐free culture conditions, we were able to support long‐term maintenance of the bone and immune compartments over defined culture periods (up to 42 days for 3D BOAC cultures) within a dynamic microfluidic environment. As proposed by Bourgine et al. [18]. an ideal BM model must combine cellular diversity, physiological architecture, and dynamic perfusion. Our BOAC meets these critical requirements and enables real‐time monitoring of the soluble environment. Continuous perfusion ensures dynamic exchange of metabolites, while the 3D architecture of the scaffolds allows the formation of the bone‐specific microenvironments, including relevant cell types and reticular connective tissue. A central advantage of our BOAC system is its ability to recapitulate key features of functional bone remodeling with preservation of adult immune cell complexity. The immune profile of the adult BM is shaped by lifelong antigen exposure and harbors all kinds of mature memory immune cell types [3, 39]. These antigen‐experienced immune cells include memory T and B cell populations that are capable to generate rapid cytokine responses when stimulated. Their distinct function is critical for translational applications in bone research, but remains largely unaddressed in existing pre‐clinical models [4, 18, 40]. In contrast to femoral head biopsies, which show a markedly reduced cytokine response to Con A treatment after 14 days of in vitro culture, PBMCs within the BOAC maintained a pronounced cytokine response upon stimulation at this time point. This highlights the ability of our system to preserve immune cell responsiveness at defined time points during in vitro culture. While the applied stimulation approach primarily addresses T cell activation and does not capture the full spectrum of immune functionality, it demonstrates that immune cells maintained within the BOAC system retain the capacity to respond to a defined polyclonal stimulus. This feature is particularly relevant for studying osteoimmune interactions under controlled in vitro conditions [41, 42]. All cell types in our BOAC culture originate from the same donor, a unique characteristic to minimize allogeneic effects that could alter cellular behavior. In combination with the use of human cancellous bone scaffolds and optimized xeno‐free media, this approach reduces cross‐species variability and enhances physiological relevance. Together, this integrated design enables the direct investigation of human osteoimmune mechanisms under near‐physiological conditions.

Our 3D BOAC system supports the differentiation of human BM and blood monocytes into functional osteoclasts. While many in vitro bone models claim to generate mature osteoclasts based on TRAP or cathepsin K expression, these markers are insufficient to confirm resorptive capacity, as they are also expressed by macrophages [43, 44, 45]. The detection of CTX‐I as a degradation product of collagen type I provides a widely used biochemical marker of osteoclastic activity. CTX‐I, together with P1NP are well‐established clinical markers in the circulation for bone remodeling, and alterations in their ratio reflect imbalances in bone turnover [46, 47]. In our in vitro system, P1NP measurement was limited due to its high basal concentration, which presumably originated from the human serum in our culture media. Nevertheless, in the more complex cellular environment of the bone biopsy model, P1NP levels remained distinguishable from the medium background (Figure S2). This observation highlights a general challenge in applying bone formation markers in fully xeno‐free in vitro systems and underscores that the choice of culture conditions must be carefully matched to the specific research question. We observed *de novo* soft tissue deposition by µCT analysis after 34 days, indicating active ECM formation, which has rarely been reported in comparable studies. Previous studies have emphasized the importance of ECM deposits in 3D cultures, considering their absence a fundamental limitation of conventional biomaterials [48]. Therefore, the formation of reticular connective tissue is an important aspect of our BOAC model. As a central structural element of the BM matrix, it contributes to the preservation of immune cells.

**TABLE 1:** Primer sequences.

Housekeeping			
**Name**	**Symbol**	**Primer**	**Sequence**
Elongation factor 1‐alpha 1	EEF1A	Left Primer	AACACAGGTGTCGTGAAAAC
	EEF1A	Right Primer	AAGACCCAGGCATACTTGAA
RPL13A protein	RPL13A	Left Primer	CCTGGAGGAGAAGAGGAAAGAGA
	RPL13A	Right Primer	TTGAGGACCTCTGTGTATTTGTCAA
Glyceraldehyde‐3‐phosphate dehydrogenase	GAPDH	Left Primer	AGTCAACGGATTTGGTCGTA
	GAPDH	Right Primer	CTGGAAGATGGTGATGGGAT
Actin, cytoplasmic 1	ACTB	Left Primer	GGACTTCGAGCAAGAGATGG
	ACTB	Right Primer	AGCACTGTGTTGGCGTACAG
Matrix			
**Name**	**Symbol**	**Primer**	**Sequence**
Decorin	DCN	Left Primer	CTGTGGCAAATTCCCGGAT
	DCN	Right Primer	GCACTTTGTCCAGACCCAAA
Fibronectin 1	FN1	Left Primer	CAGCCAGTAGCTTTGTGGTC
	FN1	Right Primer	GCATCAGGCGCTGTTGTTT
Collagen I alpha‐2(I) chain	COL1A2	Left Primer	AGCCGGAGATAGAGGACCAC
	COL1A2	Right Primer	GGCCAAGTCCAACTCCTTTT
Collagen III alpha‐1(III) chain	COL3A1	Left Primer	CCTGGAAGAGATGGAAACCC
	COL3A1	Right Primer	TCGATGTCCTTTGATGCCAG
Osteonectin	SPARC	Left Primer	GCAGAGGTGACTGAGGTATC
	SPARC	Right Primer	TTGTTGTCATTGCTGCACAC

Unlike previous studies focusing primarily on hematopoietic or pluripotent stem cells [49, 50], our approach maintained multiple immune cell subsets, including monocytes, T cells, and B cells, over a time course of up to 42 days. These populations remained stable through interaction with stromal and bone cells, without the need for targeted stimulation with cytokines, specialized culture media, or other external stimuli. While many studies emphasize the importance of immune cells in BM cultures, most efforts have focused on progenitor cells [51], single mature cell types such as plasma cells [13], BMMNCs on hydroxyapatite scaffolds [52], or immature immune cells from animal sources [14]. The Con A stimulation assays further demonstrate that immune cells preserved in the BOAC system can still respond to specific stimuli, and that T cell‐associated cytokine responses can be measured in our dynamic system [53, 54, 55]. Such T‐cell‐specific responses are particularly relevant for osteoimmunology [41], an underexplored research field, especially in the context of adult immune cell interactions in bone homeostasis [56].

We also observed differences in the composition of immune cells between BM‐based and PB‐based models, highlighting the influence of the initial seeding strategy on the resulting cellular microenvironment. BM‐BOAC primarily preserved myeloid progenitors and macrophages, which suggests a niche more similar to the natural BM environment. In contrast, a higher abundance of adaptive immune cells in PB‐BOAC may indicate a shift towards peripheral immune dynamics. To further strengthen the interpretation of the single‐nucleus RNA‐seq data, publicly available human bone marrow single‐cell RNA‐seq datasets were incorporated as a comparative reference (Figure S1). Although differences in sequencing modality (single‐nucleus vs. single‐cell, fixed vs. fresh) should be considered, this approach enables contextual validation of the identified cell populations within the broader landscape of human bone marrow biology. The observed overlap in major cell types (e.g., lymphoid and myeloid lineages) supports the biological relevance of the BOAC model, while the analysis remains primarily qualitative due to the limited sample size. Future studies will require additional donors in order to systematically evaluate donor‐to‐donor variability. In our BOAC system, we functionally tested immune cells from multiple human donors, capturing their inherent heterogeneity, which is an important factor for translational research. However, the absence of early B cells, erythrocytes, and endothelial cells in both BOAC setups – despite their presence in ex vivo biopsies – underscores a key limitation in recapitulating hematopoietic and vascular complexity.

The lack of endothelial cells indicates that the current BOAC configuration does not include a fully established vascular niche, which plays a central role in regulating hematopoiesis and immune cell trafficking in native bone marrow [5, 6]. Consequently, the model is not yet suited to study vascular‐dependent processes such as immune cell recruitment or vascular‐associated bone pathologies. Moreover, early B cell populations were not maintained in the BOAC cultures, suggesting that the system does not yet support the full spectrum of hematopoietic differentiation [39, 51]. Approaches to model vasculature, immune competence, and lymphatic vasculature have been described recently and provide an avenue for future optimization of our BOAC model [36, 57].

Dynamic medium flow is critical to maintain immune cell populations and to support bone remodeling, especially in our dense 3D model. It improves mass transport by facilitating the exchange of nutrients and metabolites and by preventing the formation of diffusion‐limited regions. While gas‐phase conditions (temperature and CO_2_) were controlled at the incubator level, oxygen concentrations within the 3D scaffold were not directly measured and are governed by convective and diffusive transport processes within the system. Based on the measured perfusion rate and scaffold microarchitecture, interstitial flow velocities fall within the low µm/s range, corresponding to shear stresses in the µPa range for culture medium and approaching the sub‐Pa range when marrow‐like effective viscosity [34] is considered. Under these conditions, convective transport ensures continuous medium renewal, while diffusion enables efficient local distribution within the pore space. Such low interstitial flows are characteristic of the trabecular bone marrow environment and are sufficient to influence gene expression and cellular behavior [15, 24, 34, 58]. Thus, the quantitative characterization of flow velocity and shear stress provides a physical framework for interpreting the improved immune cell retention and bone remodeling observed under dynamic culture conditions. In our system, controlled flow conditions were associated with sustained bone remodeling activity and better maintenance of immune cell populations compared to static conditions [15, 24, 34]. Our BOAC model remained viable by perfusion for up to 42 days, outperforming most conventional static cultures in terms of integrity and functionality. However, our current model lacks extrinsic mechanical forces, which play an important role in bone remodeling [59, 60, 61]. In addition, the absence of direct measurements of oxygen gradients represents a limitation of the current study. Thus, future studies should include cyclic mechanical stimulation and in situ sensing approaches to further approximate physiological bone dynamics.

Temperature also played a critical role in optimizing cell differentiation and maintenance. While 37°C promoted MSC/OB differentiation and monocyte‐derived osteoclastogenesis, 33°C facilitated immune cell maintenance and inhibited overpopulation by stromal cells. To leverage the advantages of both conditions, we first established the bone compartment with bone‐forming OBs and resorbing OCs at 37°C. Subsequently, we introduced immune cells in the system and shifted the temperature to 33°C to achieve long‐term homeostasis of all cell types.

Bone metabolism markers OPG and OPN progressively increased, while pro‐inflammatory cytokine levels such as IFN‐γ and TNF‐α remained stable and did not indicate a strongly pro‐inflammatory environment. OPG and OPN are opposing regulators of the bone remodeling process and served as surrogate markers for an active bone compartment. Other BOAC models have investigated the OPG/ RANKL axis, yet few have examined both factors longitudinally within the culture environment [62]. Integrating OPG and OPN monitoring into our BOAC approach enables the assessment of bone tissue formation dynamics. Notably, certain cytokines related to peripheral inflammation, such as IL‐6 and IL‐4, were detected in our system, but these are also essential for BM development and myeloid cell recruitment [63]. Stable levels of M‐CSF, VEGF‐A, and other regulators further support the functional integrity of our BOAC environment [64, 65].

Our results indicate the importance of the sequential seeding strategies and reveal the complex interactions between the different cell populations. MSC/OB‐derived factors inhibited osteoclastic resorption, whereas osteoclast‐secreted factors had less effect on osteogenic activity. This is in line with the physiological process and reflects known asymmetries in bone turnover. Sequential seeding also enabled the long‐term preservation of functional, mature immune cells, a feature lacking in many existing models. The viability of immune cells is maintained via paracrine factors and direct cell‐to‐cell contact with stromal cells. This highlights the importance of reproducing the complexity of the BM niche as accurately as possible.

To our knowledge, the BOAC model presented one of the first autologous, xeno‐free human 3D system that combines functional osteogenesis, bone resorption, immune cell maintenance, and a balanced cytokine profile on a native bone scaffold within a standardized perfusion platform. It allows for the modeling of patient‐specific cellular heterogeneity and supports long‐term studies of adult immune biology. The modularity of our system enables its adaptation to other human bone pathologies, including osteoporosis, multiple myeloma, and age‐related immune dysfunction.

## 4 Conclusions and Future Directions

Our BOAC system meets the key requirements for complex in vitro bone and bone marrow models. Its physiological relevance is enhanced by the use of human cancellous bone scaffolds and autologous primary cells. The complexity of human bone architecture cannot be synthetically reproduced so far, but native bone matrix, together with minimally expanded BM precursor cells, enables the self‐organization of tissue in vitro. We observed reticular soft tissue formation, active bone metabolism, and a soluble environment that promotes homeostasis, without excessive bone resorption, inflammation, or cell death. Despite the limitations of our system, its modular architecture allows flexible adaptation to diverse research questions and clinical indications. Future work will focus on adapting the system using iPSC‐derived cells, which will enable genetic modification and expand the system's applicability to congenital conditions such as osteopetrosis and other rare diseases.

## 5 Methods

### 5.1 Human Tissue Sources and Bone Scaffold

Blood samples, primary human bone marrow (BM) aspirates, and femoral head tissue biopsies were obtained from patients undergoing primary hip arthroplasty at Charité Universitätsmedizin Berlin. All patients provided informed consent, and procedures were approved by the local ethics committees (EA099/10, EA2/089/20). Cancellous bone scaffolds were obtained from the tissue bank at Charité Universitaetsmedizin Berlin. These scaffolds were pre‐cut into cylindrical shapes to fit a 96‐well format, with dimensions of 6 mm in diameter and 10 mm in height. The bone scaffolds were devoid of organic material and cells.

### 5.2 Femoral Head Tissue Biopsies

Femoral head biopsies were prepared from femoral heads obtained from patients undergoing primary hip arthroplasty. Cylindrical bone explants measuring approximately 10 mm in height and 6 mm in diameter were extracted from the femoral head using a biopsy punch. The explants were cultured in a medium consisting of 50% α‐MEM Eagle (PAN‐Biotech, Aidenbach, Germany) and 50% X‐VIVO 15 (Lonza, Basel, Switzerland), supplemented with 10% human AB serum (hAB), 1% penicillin/streptomycin (P/S; Bio&SELL, Feucht, Germany), and 1% GlutaMAX (Gibco, Thermo Fisher Scientific, Massachusetts, USA).

### 5.3 Cell Isolation and Expansion

Unless otherwise specified, all cells were cultured under standard conditions (37°C, 5% CO_2_, humidified atmosphere) in the medium formulations listed below. The cells were cultured for a maximum of three passages (P), and all experiments were performed with cells in P2–P3. To ensure culture quality, all cultures were routinely tested for mycoplasma contamination every two weeks using MycoAlert Mycoplasma Detection Kit (Lonza, Basel, Switzerland). Only cultures confirmed to be mycoplasma‐negative were used for the experiments.

### 5.4 Bone Marrow Mononuclear Cell (BMMNC) Isolation from BM Aspirates

The BM aspirates were filtered through a 100 µm cell strainer, mixed with an equal volume of sterile Dulbecco's Balanced Salt Solution (DPBS) (Thermo Fisher Scientific, Waltham, MA, USA), and separated on a Ficoll gradient. The interphase was collected, washed, and the cells were counted using a CASY counter (OLS, Bremen, Germany).

### 5.5 Isolation of Primary Human Mesenchymal Stromal Cells (MSCs) from BM Samples

MSCs were isolated and characterized as described previously [66, 67]. The BM sample was transferred to a falcon tube and mixed in equal parts with sterile DPBS. The tube was inverted several times and passed through a 100 µm cell strainer to remove coagulated blood and micron‐sized bone particles. Cancellous bone pieces were extracted and stored in DPBS for osteoblast isolation. The mixture was centrifuged at 400 x g for 10 min at RT, and the lipid layer was discarded. Cell suspension, including BMMNCs, was carefully layered on Histopaque 1 077 (Sigma–Aldrich, Missouri, USA), a commercial solution for blood cell isolation. The cells were centrifuged at 400 x g for 30 min at RT without a break. The interphase was collected and pooled. Cell numbers were counted using a CASY cell counter (OLS OMNI Life Science, Bremen, Germany). Pre‐warmed expansion medium (EM; DMEM low glucose with 10% FBS [Biochrom, Berlin, Germany]), 5% Glutamax [Gibco/Thermo Fisher Scientific, Massachusetts, USA], 1% PenStrep [Bio&SELL, Feucht, Germany]) was added to the cell suspension and seeded into cell culture flasks at a density of 0.5 × 10^6^ cells per flask. EM was changed every other day. For further expansion, cells were split at 80% confluency using 0.05% trypsin with EDTA (Thermo Fisher Scientific, Massachusetts, USA).

### 5.6 Isolation of Primary Human Osteoblasts (OBs) from Cancellous Bone

OBs were isolated as previously described [68]. Briefly, cancellous bone pieces were washed in DPBS by shaking and vortexing in a falcon tube until they appeared completely clean, exhibiting a white color. The bone was then finely chopped with a scalpel. These pieces were transferred into a culture flask and filled with prewarmed EM. OB migrates out of the bone pieces and adheres to the culture flask. The cells were incubated for 3–4 weeks to reach 80% confluency.

### 5.7 Isolation of Primary Human Monocyte from BMMNCs or PBMCs

After isolation of BMMNCs or peripheral blood mononuclear cells (PBMCs) from primary BM or blood, enrichment of classical (CD14^++^ CD16^−^), nonclassical (CD14^+^ CD16^++^), and intermediate (CD14^++^ CD16^+^) monocytes was performed using the Pan monocyte isolation kit (Miltenyi Biotec, Bergisch Gladbach, Germany). Isolation was performed according to the manufacturer's instructions. In brief: First, the cell number was determined, and magnetic labeling of the cell suspension was manual performed using the appropriate amounts of FcR Blocking Reagent, Biotin‐Antibody Cocktail, and Anti‐Biotin MicroBeads. Labeled cell suspension was subsequently applied to the magnetic column and washed three times with 3 mL MACS buffer. MACS buffer contained 0.5% BSA (Sigma–Aldrich, Missouri, USA) and 2 mm EDTA (Thermo Fisher Scientific, Waltham, MA, USA) in DPBS (Thermo Fisher Scientific, Waltham, MA, USA), pH 7.2. The negative cell fraction, which consisted of the unlabeled monocytes, was collected and seeded directly for OC differentiation.

### 5.8 Basic and T Cell Immune Cell Characterization of Primary Material Using Flow Cytometry

Basic immune cell staining for flow cytometry was performed using DURAClone IM Phenotyping BASIC (B53309, Beckman Coulter, Washington, D.C., USA) according to their protocol. DURAClone is a lyophilized, precoated 8‐monoclonal‐antibody panel for fast clinical downstream analysis. For basic immune subset identification, the following antibodies included in the panel were used: mouse anti‐CD16‐FITC, mouse anti‐CD56‐PE, mouse anti‐CD19‐ECD, mouse anti‐CD14‐PC7, mouse anti‐CD4‐APC, mouse anti‐CD8‐A700, mouse anti‐CD3‐APC‐A750, and mouse anti‐CD45‐KrO (Beckman Coulter, Washington, D.C., USA). For additional staining, monoclonal mouse anti‐CD279‐PC5.5 (B36123, Beckman‐Coulter, Washington D.C., USA) was added at a dilution of 1:25 for PBMCs and 1:50 for BM. 100 µL of undiluted BM aspirate, or 1 × 10^6^ PBMCs were added to one DURAClone tube and incubated at RT for 15 min. VersaLyse solution (Beckman Coulter, Washington, D.C., USA) was then added, and the sample centrifuged and washed twice before resuspension in 200 µL buffer (1x PBS with 5% v/v of fetal bovine serum, 2 mm EDTA, and 2 mm sodium azide). Flow cytometry data were acquired using a NAVIOS flow cytometer (Beckman–Coulter, Washington D.C., USA) and analyzed with Kaluza software (Beckman Coulter, Washington, D.C., USA). The gating strategy followed the manufacturer's recommendations [69] and is shown in Figure S3 [69].

### 5.9 Osteoclast Differentiation

OCs were differentiated according to an adapted version of the protocol by Rössler et al. [30]. In brief, 2.3 × 10^5^ PB‐Monocytes or 3.1 × 10^5^ BM‐Monocytes per cm^2^ cell culture vessels were seeded in α‐Minimum Essential Medium Eagle (α‐MEM Eagle, PAN‐Biotech, Aidenbach, Germany) with 10% heat‐inactivated human serum (hi‐hAB, Sigma–Aldrich, Missouri, USA), 1% Pen/Strep (Bio&SELL, Feucht, Germany), 1% Glutamax (Gibco/Thermo Fisher Scientific, Massachusetts, USA) and 50 ng/ml recombinant human macrophage colony‐stimulating factor (rhM‐CSF, R&D systems, Minnesota, USA). Three days after seeding, half of the culture medium was replaced with fresh OC medium (α‐MEM Eagle, 10% hi‐hAB, 1% Pen/Strep, 1% Glutamax, 100 ng/ml rhM‐CSF, 100 ng/ml recombinant human soluble receptor activator of nuclear factor‐κB ligand (rhsRANKL, PeproTech/Thermo Fisher Scientific, MA, USA). The cells were cultured for an additional 11 or 18 days. Every 2 to 3 days, half of the medium was replaced with fresh OC medium.

### 5.10 Osteogenic Differentiation

After isolation and expansion of human MSCs and human OBs, the cells were detached using 0.05% trypsin with EDTA (Thermo Fisher Scientific, MA, USA) and seeded with a cell density of 12,8 × 10^3^ MSCs/OBs per cm^2^ in expansion medium (α‐MEM Eagle with 10% hi‐hAB, 1% Pen/Strep, 1% Glutamax). The next day, the expansion medium was completely replaced by osteogenic medium (α‐MEM Eagle with 10% hi‐hAB, 1% Pen/Strep, 1% Glutamax, 50 µm L‐Ascorbic acid 2‐phosphate sesquimagnesium salt hydrate (Sigma–Aldrich, Missouri, USA), 10 mm β‐Glycerolphosphate disodium salt hydrate (Sigma–Aldrich, Missouri, USA), 0.1 µm Dexamethasone‐water soluble (Sigma–Aldrich, Missouri, USA). Culture medium was completely replaced with fresh OM medium every three to four days. Osteogenic culture was stopped after 10 to 21 days of stimulation, depending on donor performance.

### 5.11 Coculture Systems

Indirect cocultures were established by exchanging conditioned media between OB/MSC and OC cultures. For direct cocultures, OCs are located on a cortical bovine bone slice (boneslices.com, Jelling, Denmark) in a 1 µm pore size transwell (Corning, New York, USA) hanging in a 24‐well of a 24‐well tissue culture plate (Corning, New York, USA). OCs are spatially separated from MSCs and OBs, which are located at the bottom of the 24‐well. All cells share the same medium. On the bone slice, either 0.75 × 10^5^ PB‐monocytes or 1 × 10^5^ BM‐monocytes were seeded and differentiation as described in a previous section. After 14 days of OC differentiation, 1.35 × 10^4^ MSCs and 4.5 × 10^3^ OBs were mixed and seeded on the bottom of the 24‐well and stimulated osteogenically for 14 days as described in a previous section. After four weeks of culture, functional readouts such as lectin staining, CTX‐I quantification and AR staining were performed.

### 5.12 BOAC Culture (Optimized Set‐Up)

For the cultivation of the bone chips, the HUMIMIC Chip2 system from TissUse GmbH (Berlin, Germany) was used. The microfluidic culture setup is based on the work of Sieber et al., 2018 [15] and was modified for the purposes of this study. All experiments were conducted in a temperature‐ and CO_2_‐controlled incubator (37°C or 33°C, 5% CO_2_), ensuring stable gas‐phase conditions throughout the cultivation period. Cultivation was carried out over a total of six weeks with a dynamic flow of 0.5 Hz at ± 500 mbar, generating a unidirectional pulsatile flow within the microfluidic system. Prior to the biological experiments, medium perfusion characteristics of the chip system were quantified by particle image velocimetry (PIV) using a bead suspension as tracer particles. Flow velocities were recorded at defined regions of interest within the microfluidic circuit over one pump cycle and used to calculate the average volumetric flow rate. These measurements showed an average flow rate of 6.3 ± 0.8 µL/min per circuit. Comparable flow rates were measured in chips with and without bone scaffolds, indicating that scaffold insertion did not substantially alter overall medium perfusion and that flow conditions were reproducible across experimental setups. Each compartment held a volume of 400 µL medium. A half medium change was performed every 2–3 days during the first two weeks, followed by a half medium change every 3–4 days until the end of the culture. A human decellularized cancellous bone cylinder (height: 10 mm, width: 6mm) from the tissue bank of the Charité Universitätsmedizin was used as a scaffold. First, BM monocytes or PB monocytes were seeded at a density of 1.5 × 10^6^ cells/scaffold after magnetic isolation as previously described. These were differentiated into OCs within 14 days according to the protocol described above, with increased cytokine concentrations of rhM‐CSF and rhsRANKL to 150 ng/mL. After two weeks, 2.25 × 10^5^ MSCs and 0.75 × 10^5^ OBs were mixed per scaffold, seeded onto the OC‐containing scaffold, and osteogenically differentiated under dynamic conditions for an additional 14 days, as previously described. In the last step, 2 × 10^6^ BMMNCs or PBMCs were added per scaffold. These scaffolds were cultured for a further 14 days in 50% α‐MEM Eagle (PAN‐Biotech, Aidenbach, Germany)/ 50% X‐vivo15 (Lonza, Basel, Switzerland) with 10% hAB and 1% P/S (Bio&SELL, Feucht, Germany) and 1% Glutamax (Gibco/Thermo Fisher Scientific, Massachusetts, USA). Supernatants were collected at each medium change to monitor LDH, BALP and CTX‐I. In addition, the scaffold was scanned using µCT for co‐registration before and after culture.

### 5.13 Concanavalin A (Con A) Treatment

Femoral head punches or human bone scaffolds colonized with 2 × 10^6^ PBMCs were cultured for 7 or 14 days in a medium consisting of 50% α‐MEM Eagle (PAN‐Biotech, Aidenbach, Germany) and 50% X‐VIVO 15 (Lonza, Basel, Switzerland), supplemented with 10% human AB serum (hAB), 1% penicillin/streptomycin (P/S; Bio&SELL, Feucht, Germany), and 1% GlutaMAX (Gibco, Thermo Fisher Scientific, Massachusetts, USA). Following the culture period, samples were stimulated with 50 µg/mL Concanavalin A (Merck, Darmstadt, Germany) for 24 h. Supernatants were collected immediately before stimulation and after 24 h for subsequent cytokine analysis.

### 5.14 CyQuant Cell Proliferation Assay

The CyQuant proliferation assay kit (Thermo Fisher Scientific, Waltham, MA, USA) was used according to the manufacturer's instructions. Following a freeze–thaw cycle, cells were incubated with the reagent for 5 min at room temperature (RT) and analyzed using a plate reader (Tecan, Männedorf, Switzerland) at Ex/Em = 480/520 nm.

### 5.15 PrestoBlue Assay

PrestoBlue (Thermo Fisher Scientific, Waltham, MA, USA) was used as instructed: Cells were incubated with PrestoBlue (diluted 1:10) for 1 h at 37°C. Fluorescence was measured at Ex/Em = 560/590 nm using a plate reader (Tecan, Männedorf, Switzerland).

### 5.16 Bone‐Specific Alkaline Phosphatase (BALP) Activity (in 2D and 3D)

In 2D, BALP activity was assessed by measuring its enzymatic activity according to the manufacturers protocol. In brief: The substrate 4‐nitrophenyl phosphate (p‐NPP, Sigma–Aldrich, Missouri, USA) was added directly to adherent cells in each well. BALP catalyzed the hydrolysis of p‐NPP to 4‐nitrophenol, resulting in a yellow‐colored product. The absorbance of the reaction was measured photometrically using a plate reader (Tecan, Männedorf, Switzerland). In 3D, BALP was detected in cell culture supernatant using human BALP Sandwich ELISA (AFG Scientific, Northbrook, IL, USA). The assay was performed according to the manufacturer's instructions. In brief: Samples were diluted 1:5, and 50 µL of each sample and the standard concentrations were applied to the 96‐well ELISA plate. The plate was incubated for 30 min at 37°C. It was then washed 5 times with 1x wash solution, and 50 µL Chromogen solution A and 50µL Chromogen solution B were added to each well. The plate was incubated for 15 min at 37°C, and the reaction was stopped with 50 µL stop solution per well. Absorbance was measured at 450 nm with reference at 650 nm. BALP concentration was calculated using a standard linear equation.

### 5.17 Procollagen I N‐Terminal Propeptide (P1NP) Quantification

P1NP concentration was determined in cell culture supernatant using a human sandwich ELISA (Biozol, Hamburg, Germany) according to the manufacturer's instructions. All reagents were equilibrated to room temperature for at least 60 min before use, and the wash buffer was prepared according to the protocol. Samples were diluted 1:10 in PBS in a separate 96‐well plate. Detection reagents A and B were prepared by centrifuging the vials briefly and diluting each reagent 1:100 with the corresponding assay diluent. A two‐fold serial dilution of the standard was prepared immediately prior to use. Subsequently, 100 µL of standards and pre‐diluted samples were added to the antibody‐coated microplate and incubated for 1 h at 37 °C. After removal of the liquid without washing, 100 µL of Detection Reagent A was added to each well, followed by incubation for 1 h at 37 °C. Wells were then washed three times with 300 µL wash buffer per well, after which 100 µL Detection Reagent B was added and incubated for 30 min at 37 °C. The plate was washed five times with 300 µL wash buffer per well. Next, 90 µL substrate solution was added to each well and incubated for 10 min at 37 °C protected from light. The reaction was stopped by adding 50 µL stop solution to each well, resulting in a color change from blue to yellow. Absorbance was measured immediately at 450 nm using a microplate reader (Tecan, Männedorf, Switzerland).

### 5.18 Alizarin Red (AR) Staining and Quantification

AR staining was performed as described previously [66]. Briefly, after osteogenic cultivation, cells were washed once with 1× DPBS and fixed with 4% paraformaldehyde (PFA; Roti Histofix, Roth, Karlsruhe, Germany) for 10 min at room temperature (RT). Fixed cells were washed twice with 1× DPBS and could be stored in 1× DPBS at 4 °C until further processing. For quantitative analysis, Hoechst staining was performed prior to AR staining. Background fluorescence was recorded in 1× DPBS at Ex/Em = 364/460 nm using a plate reader (Tecan, Männedorf, Switzerland). Cells were then incubated with 28.6 mm Hoechst (bis‐Benzimid H 33258; Sigma–Aldrich, Missouri, USA) diluted 1:1 000 in 1× DPBS for 10 min in the dark at RT, washed three times with 1× DPBS, and fluorescence was measured again at Ex/Em = 364/460 nm. After Hoechst measurement, cells were washed once with ddH_2_O and incubated with AR‐S solution (Sigma–Aldrich, Missouri, USA) for 10 min at RT. Excess dye was removed, and cells were washed at least three times for 5 min each until no visible discoloration of the matrix remained. Stained wells were dried for 24 h, and macroscopic as well as microscopic images were acquired at 4× and 10× magnification. For quantitative analysis of mineralized matrix, wells were incubated on an orbital shaker with cetylpyridinium chloride (CPC; Roth, Karlsruhe, Germany) until complete dye dissolution. The absorbance of CPC solutions was measured at 562 nm using a plate reader. Final AR values were normalized to Hoechst fluorescence

### 5.19 Lactate Dehydrogenase (LDH) for Cytotoxicity Assessment in Supernatant

LDH is an enzyme released from the cytoplasm of damaged cells, making it a suitable marker for quantifying cell death and lysis. The enzyme's activity in the supernatant correlates with the number of damaged or dead cells. This enzymatic activity, measured through a two‐step reaction coupled with a catalyst, is utilized in the commercially available cytotoxicity detection kit from Roche (Basel, Switzerland). The assay involves mixing the culture supernatant with dye and a catalyst. The absorbance is then detected using a plate reader (Tecan, Männedorf, Switzerland) at 500 nm. The samples can be stored at 4°C up to one week and measured at once.

### 5.20 C‐terminal Telopeptides of Type I Collagen (CTX‐I) Quantification

Human CTX‐I was detected in cell culture supernatant using human CTX‐I Sandwich ELISA (Immunodiagnostic systems, Frankfurt, Germany). The assay was performed according to the manufacturer's instructions. In brief: Samples were diluted 1:5, and 50 µL each of the sample, standard, and positive control was applied to the 96‐well ELISA plate. Next, 150 µL of antibody solution was added, and the plate was incubated for 2 h at RT and 300 rpm. The plate was then washed 5 times with 1x wash buffer, and after adding 100 µL substrate solution, it was incubated again for 15 min at RT in the dark at 300 rpm. The reaction was stopped with 100 µL stop solution, and absorbance was measured at 450 nm with reference at 650 nm. CTX‐I concentration was calculated using a standard linear equation.

### 5.21 Phosphate Quantification

Phosphate levels in cell culture supernatants were determined using a colorimetric phosphate assay kit (Abcam, Cambridge, Great Britain). The assay was performed according to the manufacturer's instructions. In brief: First, samples were diluted with ddH2O, sample concentration was dependent on the type of culture (1:100 for expansion cultures or 1:800 for osteogenic cultures). In the next step, 200 µL of diluted samples and standards were incubated with 30 µL phosphate reaction mix for 30 min in the dark. After incubation, absorption at 650 nm was measured using a plate reader (Tecan, Männedorf, Switzerland). Phosphate concentration was calculated using a standard linear equation.

### 5.22 Resorption Pit Assay on Osteo Surface

OC were differentiated on Osteo Assay Surface Strips (Corning, New York, USA, #CLS3989) using the protocol described above. Following cultivation, the cells were washed twice with DPBS (Thermo Fisher Scientific, Waltham, MA, USA) and removed by treatment with 150 µL/cm^2^ of 5% sodium hypochlorite (Merck, Darmstadt, Germany) for 5 min at RT. The wells were then washed twice with distilled water and air‐dried for 4 h at room temperature. Resorption pits were visualized using a DM IL LED inverted microscope (Leica, Wetzlar, Germany).

### 5.23 Lectin Staining and Imaging

After OC differentiation, the cell culture medium was removed, and the bone slices were washed 3 times with distilled water and incubated with distilled water for at least 30 min at RT. After aspiration of the distilled water, the bone slices were wiped with a paper tissue to remove residual cell debris. The bone slices were washed once with DPBS (Thermo Fisher Scientific, Waltham, MA, USA) and once with distilled water and dried using a disposal KIMTECH wipe (Roth, Karlsruhe, Germany). The bone slices were incubated with 0.01 mg/mL Wheat Germ Agglutinin‐Alexa Flour 488 (Thermo Fisher Scientific, Waltham, MA, USA) in 1% BSA (Sigma–Aldrich, Missouri, USA) in DPBS for 1 h on an orbital shaker in the dark. After washing three times with DPBS, the fluorescence intensity on both sides of the bone slices was measured using a plate reader (Tecan, Männedorf, Switzerland) at Ex/Em = 495/519 nm. If required, the bone slices were imaged using a Live Cell Microscope Incubator (Okolab–Leica, Wetzlar, Germany) at 5x magnification, and a mosaic image was created. The stained bone slices were stored in DPBS at 4°C.

### 5.24 OC Immunofluorescence Staining

The cells were fixed with 4% paraformaldehyde (PFA) (Roth, Karlsruhe, Germany) for 10 min at RT and washed three times with DPBS (Thermo Fisher Scientific, Waltham, MA, USA). For tartrate‐resistant acid phosphatase (TRAP) staining, the cells were incubated for 20 min at room temperature in the dark with a freshly prepared TRAP solution containing 5 ml TRAP buffer (40 mm sodium acetate and 10 mm sodium tartrate at pH 5.0; Merck, Darmstadt, Germany), 50 µL naphthol AS‐MX phosphate solution (1 mg dissolved in 100 µL N,N‐dimethylformamide; Sigma–Aldrich, Missouri, USA) and 3 mg fast red violet LB (Sigma–Aldrich, Missouri, USA). After two washes with DPBS, the cells were permeabilized for 10 min in a solution containing 0.1% Triton X‐100 (Merck, Darmstadt, Germany) and 3% BSA (Sigma–Aldrich, Missouri, USA) in DPBS. The cells were then incubated overnight at 4 °C in the dark with Phalloidin‐Alexa Fluor 488 diluted in 3% BSA/DPBS (1:400; Invitrogen, Waltham, MA, USA). The following day, the cells were washed twice with DPBS and counterstained with DAPI (1:1 000; Sigma–Aldrich, Missouri, USA) for 10 min at RT. After two final washes in DPBS, the samples were stored in DPBS until imaging. Images were acquired using a Revolve microscope (Echo, Cerritos, CA, USA) at 10× magnification.

### 5.25 Bone‐On‐A‐Chip (BOAC) Immunohistochemistry Staining and Sample Preparation

The scaffolds for immunohistochemistry (IHC) were placed in 4% PFA (Roti Histofix, Roth, Karlsruhe, Germany) overnight. On the next day, the PFA was removed, and the scaffolds were rinsed with DPBS (Thermo Fisher Scientific, Waltham, MA, USA). Subsequently, they were cut into four pieces and placed into a 48‐well plate. Cells were permeabilized with 0.025% Triton X‐100 (Merck, Darmstadt, Germany) in TRIS‐buffered saline (TBS; Thermo Fisher Scientific, Massachusetts, USA) for 30 min at RT. Samples were washed with 1% bovine serum albumin (BSA; Sigma–Aldrich, Missouri, USA) in TBS for 10 min, followed by a blocking step using 5% goat serum (BIOZOL, Hamburg, Germany) and 1% BSA in TBS for 30 min. For primary antibody incubation, (mouse) anti‐human RUNX2 (ab76956, Abcam, Cambridge, UK) was diluted 1:100 in Dako antibody diluent (Agilent, Santa Clara, CA, USA) and incubated overnight at 4°C. On the next day, the samples were washed three times for 5 min each with TBS containing 0.025% Triton X‐100. Secondary antibody (goat) anti‐mouse IgG (H+L)‐Alexa Flour 488 (A‐11001, Thermo Fisher Scientific, Massachusetts, USA) was diluted 1:1 000 in TBS with 1% BSA and applied together with Phalloidin–Alexa Fluor 546 (1:100, Thermo Fisher Scientific) for 1 h at RT. Following three additional washing steps with TBS/0.025% Triton X‐100, samples were rinsed twice with ddH_2_O. Nuclear staining was performed using DAPI (Invitrogen, Waltham, MA, USA) 1:1 000 in PBS for 15 min at RT, followed by two more washes with ddH_2_O. Finally, DPBS was added before imaging. Samples were analyzed using a TCS SP5 multiphoton microscope (Leica, Wetzler, Germany).

### 5.26 Cathepsin K/ Hematoxylin Staining

BOAC and ex vivo bone biopsy samples were fixed in 4% PFA (Roti Histofix, Roth, Karlsruhe, Germany) for three days and stored in DPBS (Thermo Fisher Scientific, Waltham, MA, USA) with 1% PenStrep (Bio&SELL, Feucht, Germany). For paraffin embedding, bones were decalcified in EDTA (Thermo Fisher Scientific, Waltham, MA, USA) at 37°C for three weeks, with EDTA replaced every 3 days. Samples were dehydrated using an automatic tissue filtration machine (Leica, Wetzler, Germany) and embedded in paraffin. 5 µm – sections were cut, dried at 37°C for one day, deparaffinized in xylene (Thermo Fisher Scientific, Waltham, MA, USA), rehydrated with an alcohol gradient, and washed with DPBS. Antigen retrieval was performed in citrate buffer at 60°C for 6 h. After DPBS washes, sections were blocked in 3% BSA/DPBS for 30 min, then incubated overnight with a 1:200 dilution of anti‐Cathepsin K antibody (Proteintech, Rosemont, IL, USA). The next day, sections were washed, incubated with the secondary antibody for 1 h at RT in the dark, washed again, and counterstained with Mayer's hematoxylin (Merck, Darmstadt, Germany). Finally, sections were washed, rinsed in distilled water and mounted with Fluoromount G (Invitrogen, Waltham, MA, USA). Sections were imaged using a Zeiss Axio Scope 5 microscope with Zeiss Axiocam MRc microscope camera (Zeiss, Jena, Germany).

### 5.27 Confocal Fluorescence and Second Harmonic Imaging Microscopy (SHG)

Confocal and second harmonic microscopy were performed on a Leica SP5 II microscope equipped with a Spectra Physics Ti:Sapphire Laser (Mai Tai HP) with 100fs pulse width at 80 MHz and wavelength of 910 nm. SHG signal was detected at 450–460 nm with a 25x water immersion objective with a numerical aperture of 0.95 as described before [70, 71].

### 5.28 Scanning Electron Microscopy (SEM)

Samples were fixed onto aluminum stubs using conductive carbon tape and subsequently coated with a thin layer of gold using a JEOL JFC‐1200 sputter coater (JEOL GmbH, Germany). The coating process was carried out under an argon atmosphere at 30 mA and 8 Pa for 30 s. Imaging was performed with a JEOL JCM‐600 scanning electron microscope (JEOL GmbH, Germany) operating at an accelerating voltage of 15 kV and under high vacuum conditions. Images were captured at the magnifications specified in the respective figures.

### 5.29 Synchrotron‐Based Micro‐Computed Tomography

Synchrotron‐based micro‐computed tomography (µCT) imaging was performed at two different beamlines to visualize soft tissue structures and osteoclast (OC)‐mediated bone resorption in BOAC cultures. Visualization of OC resorption: High‐resolution µCT imaging was performed at the ANATOMIX beamline of the SOLEIL synchrotron (Paris, France) to visualize OC‐mediated bone resorption [72, 73]. Measurements were taken using a filtered white beam with a mean photon energy of around 30 keV. For each tomographic image, 4,720 projections were evenly recorded over 360°. To increase the effective field of view, the center of rotation was shifted laterally by 500 pixels (half‐acquisition mode). The exposure time was 35 ms per projection. Transmission radiographs were recorded using a Hamamatsu ORCA Flash 4.0 V2 camera (2048 × 2048 pixels) coupled to a 20 µm‐thick LuAG scintillator (Crytur, Turnov, Czech Republic) via a 10× Mitutoyo microscope lens (NA = 0.28) [74]. This resulted in an isotropic, voxel‐accurate resolution of 0.65 µm. The distance between the sample and the scintillator was approximately 45 mm. Imaging of soft tissue structures: To evaluate the soft tissue components within BOAC cultures, synchrotron µCT images were captured at the ID17 beamline of the European Synchrotron Radiation Facility (ESRF, Grenoble, France). A monochromatic X‐ray beam with an energy of 47 keV was used. The optical configuration enabled an isotropic, voxel‐accurate resolution of 3.07 µm. For each scan, 2 800 projections were recorded in half‐acquisition mode over a complete 360° rotation to cover the entire sample width. The exposure time per projection was 100ms. The volume data were reconstructed using Paganin phase retrieval, analogous to the procedure described below. Tomographic reconstruction: The tomograms collected at Anatomix and ID17 were reconstructed using the standardized data pipeline of the ANATOMIX beamline and the ID17 beamline, respectively. Prior to reconstruction, the projection data were pre‐filtered using Paganin's phase‐retrieval method. Reconstruction was employed using the PyHST2 program62 (ESRF, Grenoble, France), applying the filtered back projection algorithm.

### 5.30 mRNA Isolation and qPCR

RNA was isolated using the RNeasy Plus Mini Kit (Qiagen, Hilden, Germany). To effectively lyse RNA from the bone constructs, the scaffolds were flushed multiple times with 350 µL of RLT Plus lysis buffer and subsequently frozen in the lysate at −80°C. After thawing, the lysate was collected, and the remaining lysate within the scaffold was extracted by centrifugation at 400 x g and combined with the initial lysate. After RNA isolation according to the manufacturers protocol for total RNA isolation from animal cells, RNA yield was quantified using the nanodrop system (Thermo Fisher Scientific, Waltham, MA, USA). Isolated mRNA (750 ng of each sample) was reverse transcribed into cDNA applying the iScript cDNA Synthesis Kit (Bio‐Rad, Hercules, California, USA) according to the manufacturers protocol. Gene expression was analyzed by RT‐qPCR using the LightCycler 480 SYBR Green I Master (Roche, Basel, Switzerland) according to the manufacturers protocol using the LightCycler 480 system Master (Roche, Basel, Switzerland). Oligonucleotides (Thermo Fisher Scientific, Waltham, MA, USA) are listed in Table 1.

### 5.31 Human Cytokine Array

Cytokine concentrations were detected in the 3D BOAC supernatant using human cytokine array G5 (Raybiotech life Inc., Peachtree Corners, GA, USA). The array was prepared as described by the manufacturer, and samples were used undiluted as recommended. The array was based off the sandwich ELISA principle using a Biotin‐streptavidin detection method. Analysis of the read‐out was performed using FIJI imager.

### 5.32 Soluble Protein Analysis

Soluble protein levels in culture supernatants (TGF‐β1, TGF‐β2, TGF‐β3, FGF‐23, G‐CSF, GM‐CSF, IFN‐γ, IL‐12p70, IL‐1β, IL‐2, IL‐6, IL‐8, TNF‐α, basic FGF, soluble CD40L, Granzyme B, IL‐17A, RANKL, and VEGF‐A) were quantified using customized multiplex U‐PLEX assays (Meso Scale Diagnostics, Rockville, MD, USA) according to the manufacturer's instructions. Briefly, linker‐coupled capture antibodies were used to coat assay plates. After washing, 50 µL of standards or samples (diluted 1:2) were added and incubated for 2 h at room temperature with shaking. Following washing, detection antibodies were applied and incubated prior to a final wash step. Plates were developed using MSD GOLD Read Buffer B and immediately analyzed using the MSD detection system.

### 5.33 Multiplex Immunoassay

Cytokine concentrations in cell culture supernatants collected before and after Con A stimulation were quantified using an automated microfluidic multiplex immunoassay (Ella, Bio‐Techne, Minneapolis, MN, USA) according to the manufacturer's instructions. On the day of analysis, system performance was verified using a quality control (QC) cartridge prior to sample processing, and measurements were only continued if all QC parameters were passed. Samples and reagents were equilibrated to room temperature, while samples were thawed on ice. After thawing, samples were diluted 1:2 in the provided sample diluent. Subsequently, 50 µL of the diluted sample was loaded into each designated well of the Ella cartridge. In addition, 50 µL of undiluted QC controls were added to the respective control wells. Wash buffer was added to the reservoir wells according to the manufacturer's protocol, and the run was initiated using the SimplePlex Runner software. Cytokine concentrations were measured automatically within the microfluidic cartridge, and the results were reviewed and exported using the SimplePlex software for subsequent data analysis.

### 5.34 Single‐Nuclei RNA Sequencing

Single‐nuclei RNA sequencing was performed on single replicates of two primary BOAC systems colonized with BMMNCs (BM‐BOAC) or PBMCs (PB‐BOAC), alongside a matched donor biopsy serving as reference. Nuclei were extracted from fixed and frozen samples (10x Genomics tissue fixation protocol) by mechanical disruption into small fragments, followed by incubation at 37 °C for 10 min in dissociation buffer (1 mg/mL Liberase TH, Merck, Darmstadt, Germany, in RPMI 1 640, Gibco/Thermo Fisher Scientific, MA, USA). The suspension was filtered through a 10 µm pluriStrainer (pluriSelect Life Science, Leipzig, Germany), pelleted by centrifugation (850 × g, 5 min, RT), and resuspended in quenching buffer (10x Genomics, Pleasanton, CA, USA). Multiplexing was achieved using barcoded Chromium Human Transcriptome Probe Sets, and single‐nuclei RNA libraries were prepared with the Chromium Next GEM Single Cell Fixed RNA Sample Preparation Kit on a Chromium X instrument (10x Genomics, Pleasanton, CA, USA). Libraries were quantified, and fragment size distribution was assessed using a Qubit dsDNA HS assay (Thermo Fisher Scientific, Waltham, MA, USA) and a HS DNA Kit assay on a 2100 Bioanalyzer device (Agilent). Samples were sequenced using P3 (100 Cycles) reagents on a NextSeq 2000 system (Illumina, San Diego, CA, USA). Sequencing data was demultiplexed and converted to FASTQ format using bcl2fastq2 v2.20 (Illumina, San Diego, CA, USA), and count matrices were generated with Cellranger v7.0.0 (10x Genomics, Pleasanton, CA, USA) using reference transcriptome GRCh38‐2020‐A (10x Genomics, Pleasanton, CA, USA). Subsequent analysis was performed using Seurat v4 [75] and standard R packages. Custom filtering at the cell level was performed with a minimum of 300 UMI counts and between 250 and 9 000 distinct features (genes), a maximum of 10% mitochondrial reads, as well as a doublet score [76] below 0.2. Cluster annotation was performed based on differentially expressed marker genes from published data. Publicly available human bone marrow single‐cell RNA‐seq datasets were obtained from established repositories [77, 78] and used as a reference for comparative analysis. Data integration and visualization were performed using standard workflows in R and Seurat v5 [79], and harmony [80]. Reference datasets were mapped onto the BOAC‐derived cell type annotations to enable qualitative comparison of major cell populations. Comparative analyses were performed to assess overlap in major cell populations between BOAC‐derived single‐nucleus RNA‐seq data and public reference datasets.

### 5.35 Statistics

Quantitative data are expressed as mean ± standard error of the mean (SEM). Sample size (n) denotes biological replicates, and experiments were performed using at least three biological replicates unless otherwise indicated in the corresponding figure legends. A p‐value < 0.05 was considered statistically significant. The Shapiro–Wilk test was used to assess the normality of data distributions. For datasets exhibiting a Gaussian distribution, or when the sample size was limited (n < 5), comparisons between two groups were conducted using a two‐tailed parametric t‐test (paired or unpaired, as appropriate). For experiments involving more than two groups, one‐way or two‐way analysis of variance (ANOVA) followed by Tukey's post hoc test was applied. For datasets that did not meet the assumptions of normality, non‐parametric tests were applied: the Mann–Whitney U test for pairwise comparisons and the Kruskal–Wallis test with Dunnett's multiple comparisons test for more than two groups. All statistical analyses were performed using GraphPad Prism software (version 10.3.1). Principal component analysis (PCA) was carried out using the ClustVis platform [81].

## Author Contributions

*Conceptualization*: N.S., M.J.O., J.S., S.G.*; Data curation*: N.S., M.J.O., K.H., S.G.; *Formal analysis*: N.S., M.J.O., K.H., E.B., S.G.; *Funding acquisition*: M.J.O., S.G.; *Investigation*: N.S., M.J.O., J.S., K.H., E.B., I.M.D., M.T.; *Methodology*: N.S., M.J.O., J.S., K.H., U.K., A.W., O.K., S.R., M.S., S.G.; *Resources*: G.N.D., U.M., B.H., S.D., S.H., S.R., M.S., S.G. *Visualization*: N.S., M.J.O., E.B., K.H., S.G; *Supervision*: S.G.; *Writing – original draft*: N.S., M.J.O., S.G; *Writing – review & editing*: N.S., M.J.O., J.S., K.H., E.B, I.M.D., M.T., G.N.D., U.M., U.K., A.W., B.H., S.D., S.H., O.K., S.R. M.S., S.G.

## Funding

This study was supported by the German Research Foundation (DFG) through funding of the Collaborative Research Centre 1444 (Project ID: 427826188). N.S. & S.G. also received funding from German Federal Ministry for Research, Technology and Space (BMFTR, 161L0234B) and the European Health and Digital Executive Agency (HaDEA) through the Horizon Europe project PROTO (Grant Agreement No. 101095635). The U.K. received funding from BMFTR (161L0234A). The funders had no role in study design, data collection and analysis, decision to publish, or preparation of the manuscript.

## Ethics Approval & Patient Consent Statement

The study was approved by the local Institutional Review Board of the Charité Universitaetsmedizin Berlin (EA099/10, EA2/089/20) and all patients gave their written informed consent prior to surgery.

## Conflicts of Interest

U.M. is shareholder and CSO of TissUse GmbH, Germany.

## Supporting information




**Supporting File**: advs76409‐sup‐0001‐SuppMat.docx.

## Data Availability

The data that support the findings of this study are openly available in Mendeley Data at https://doi.org/10.17632/hc9n8gjbrp.1, reference number 1017632. The snRNA sequencing raw data is available at Gene Expression Omnibus (GEO), NCBI, https://www.ncbi.nlm.nih.gov/geo/ under Accession‐ID: GSE303776. The analysis pipeline used for snRNA‐seq processing is available on GitHub: https://github.com/SpielmannLab/Stelzer‐et‐al.‐Culturing‐human‐bone‐and‐bone‐marrow‐in‐a‐3D‐environment.
