# Supplementary material for: A 3D Human Bone and Bone Marrow‐on‐a‐Chip Model for In Vitro Bone Remodeling and Immune Cell Maintenance
Source: Adv Sci (Weinh). 2026 Jul 6;13(51):e18224. doi: 10.1002/advs.202518224 (PMC13335112; doi:10.1002/advs.202518224)
Supplement: Supplementary file 1 [file ADVS-13-e18224-s001.docx]

**Supporting Material**

A 3D Human Bone and Bone Marrow-on-a-Chip Model for In Vitro Bone Remodeling and Immune Cell Maintenance

Nina Stelzer, Melanie-Jasmin Ort, Kristian Händler, Emely Bortel, Ioanna Maria Dimitriou, Martin Textor, Georg N. Duda, Janosch Schoon, Uwe Marx, Uwe Kornak, Annika Winter, Bernhard Hesse, Stefanie Donner, Sebastian Hardt, Oliver Klein, Simon Reinke, Malte Spielmann, Sven Geißler

**Table S1: Morphological parameters of human bone scaffolds used for dynamic 3D cultures**

| Scaffold number | BV [mm^3^] | TV  [mm^3^] | BV/TV [%] | Porosity [%] | SA/BV [1/mm] | TbTh mean [µm] | PoTh mean [µm] |
| --- | --- | --- | --- | --- | --- | --- | --- |
| 1 | 18.65 | 79.51 | 23.46 | 76.54 | 12.70 | 186.00 | 694.38 |
| 2 | 18.68 | 79.51 | 23.50 | 76.50 | 12.65 | 175.20 | 667.07 |
| 3 | 19.96 | 79.51 | 25.11 | 74.89 | 11.91 | 188.32 | 658.07 |
| 4 | 17.19 | 79.51 | 21.62 | 78.38 | 13.70 | 167.67 | 627.33 |
| 5 | 22.26 | 79.51 | 27.99 | 72.01 | 10.55 | 213.20 | 718.99 |
| 6 | 15.57 | 79.51 | 19.58 | 80.42 | 15.40 | 144.82 | 647.87 |
| 7 | 17.38 | 79.51 | 21.86 | 78.14 | 12.89 | 178.44 | 727.57 |
| 8 | 21.31 | 79.51 | 26.80 | 73.20 | 11.75 | 206.00 | 619.24 |
| 9 | 18.15 | 79.51 | 22.83 | 77.17 | 12.27 | 189.77 | 759.51 |
| 10 | 12.32 | 79.51 | 15.50 | 84.50 | 17.67 | 130.98 | 719.18 |
| 11 | 16.13 | 79.51 | 20.29 | 79.71 | 16.09 | 133.65 | 538.82 |
| 12 | 18.86 | 79.51 | 23.72 | 76.28 | 13.90 | 157.99 | 598.06 |
| 13 | 16.57 | 79.51 | 20.84 | 79.16 | 12.70 | 182.14 | 787.81 |
| 14 | 15.99 | 79.51 | 20.11 | 79.89 | 13.10 | 168.50 | 798.54 |
| 15 | 19.95 | 79.51 | 25.09 | 74.91 | 10.45 | 214.33 | 745.43 |
| 16 | 15.31 | 79.51 | 19.26 | 80.74 | 13.60 | 173.56 | 808.43 |
| 17 | 17.06 | 79.67 | 21.41 | 78.59 | 16.97 | 132.90 | 523.55 |
| 18 | 19.85 | 79.67 | 24.91 | 75.09 | 11.20 | 199.68 | 644.86 |
| 19 | 11.82 | 79.67 | 14.84 | 85.16 | 19.91 | 120.18 | 584.01 |
| 20 | 17.32 | 79.67 | 21.74 | 78.26 | 15.78 | 144.23 | 665.10 |
| 21 | 12.07 | 79.67 | 15.15 | 84.85 | 20.33 | 114.72 | 602.56 |
| 22 | 15.67 | 79.67 | 19.67 | 80.33 | 14.85 | 152.80 | 669.94 |
| 23 | 21.28 | 79.67 | 26.71 | 73.29 | 12.01 | 183.84 | 558.32 |
| 24 | 14.27 | 79.67 | 17.91 | 82.09 | 13.64 | 163.11 | 950.00 |
| 25 | 9.31 | 79.67 | 11.69 | 88.31 | 15.35 | 155.18 | 1135.36 |
| 26 | 11.87 | 79.67 | 14.90 | 85.10 | 16.39 | 143.80 | 750.64 |
| 27 | 14.57 | 79.67 | 18.29 | 81.71 | 14.38 | 151.40 | 825.37 |
| 28 | 11.17 | 79.67 | 14.02 | 85.98 | 14.95 | 140.18 | 1031.81 |
| 29 | 11.37 | 79.67 | 14.27 | 85.73 | 14.74 | 151.56 | 959.35 |
| 30 | 14.72 | 79.67 | 18.48 | 81.52 | 12.37 | 171.43 | 859.94 |
| 31 | 22.61 | 79.67 | 28.38 | 71.62 | 12.07 | 180.04 | 634.42 |
| 32 | 13.98 | 79.67 | 17.55 | 82.45 | 16.07 | 139.64 | 811.13 |
| 33 | 13.22 | 79.67 | 16.59 | 83.41 | 17.45 | 127.78 | 748.38 |
| 34 | 25.67 | 79.67 | 32.22 | 67.78 | 8.83 | 233.38 | 695.14 |
| 35 | 18.06 | 79.67 | 22.67 | 77.33 | 11.94 | 179.83 | 791.59 |
| 36 | 25.33 | 79.67 | 31.79 | 68.21 | 10.76 | 199.64 | 596.42 |
| Mean | 16.82 | 79.6 | 21.13 | 78.87 | 13.93 | 166.55 | 726.51 |
| SEM | 0.66 | 0.01 | 0.83 | 0.83 | 0.43 | 4.78 | 22.81 |
| Reference | - | - | 12-30^1,2^ | 70-90^3,4^ | 10-18^2,5^ | 110-250^2,6^ | - |

BV: Mineral volume; TV: Total volume; SA: Surface Area; TbTh: Trabecular thickness; PoTh: Pore thickness.

**Table S2: Reference ranges reported for human trabecular bone microarchitecture in the femoral head and other skeletal sites**

| **Parameter** | **Reference range** |
| --- | --- |
| BV/TV [%] | 12-30^1,2^ |
| Porosity [%] | 70-90^3,4^ |
| SA/BV [1/mm] | 10-18^2,5^ |
| TbTh mean [µm] | 110-250^2,6^ |

**Supplementary Figure 1: Integration of public 10X scRNA-seq reference datasets demonstrates concordance of major immune cell populations and provides qualitative contextual validation of the BOAC system despite differences in sequencing modality and dataset composition.** (a) Cell type clusters distribution based on 10 different public references and BOAC-derived data. (b) Comparison of BOAC-derived cell type clusters (blue) with publicly available datasets (gray) reveals cell type-specific overlap across major populations. c) Quantification of cluster distribution highlights differences between datasets, including the absence of red blood cells and erythroblast/megakaryocyte populations *in vitro*, while CD4⁺ and CD8⁺ T cells as well as B cells are consistently detected across datasets, albeit at varying proportions. Due to differences in sample processing (single-nucleus versus single-cell), fixation state, and donor variability, comparisons are intended for qualitative assessment rather than quantitative interpretation of population frequencies.

**Supplementary Figure 2: Procollagen Type 1 N-terminal Propeptide (P1NP) concentrations in supernatants of bone biopsies cultured dynamically for 14 days in medium supplemented with fetal calf serum (FCS) or human AB serum (hAB).** In both culture conditions, P1NP levels remained stable over the 14-day culture period. Human AB serum contained substantially higher baseline P1NP concentrations (~7 ng/mL) compared with FCS (~1.5 ng/mL). This approximately fivefold higher background level in hAB may mask *de novo* P1NP synthesis in certain cell culture systems. Sample sizes were as follows: FCS n=4; hAB n=3.

**Supplementary Figure 3:** **Gating strategy for FACS analysis with DURAClone IM Phenotyping Basic kit.** Gating strategy for Basic characterization of immune cells, including CD45 for leukocytes, CD14 and CD16 for monocytes, CD19 for B cells, CD3 for T cells, CD4 for T helper and CD8 for cytotoxic T cells.

**References**

1.	Klinger, S., Greinwald, M., Augat, P., and Hollensteiner, M. (2022). Mechanical and morphometric characterization of custom-made trabecular bone surrogates. J Mech Behav Biomed Mater *129*, 105146. 10.1016/j.jmbbm.2022.105146.

2.	Greenwood, C., Clement, J.G., Dicken, A.J., Evans, J.P., Lyburn, I.D., Martin, R.M., Rogers, K.D., Stone, N., Adams, G., and Zioupos, P. (2015). The micro-architecture of human cancellous bone from fracture neck of femur patients in relation to the structural integrity and fracture toughness of the tissue. Bone Rep *3*, 67–75. 10.1016/j.bonr.2015.10.001.

3.	McGregor, M., Patel, S., McLachlin, S., and Vlasea, M. (2021). Architectural bone parameters and the relationship to titanium lattice design for powder bed fusion additive manufacturing. Additive Manufacturing *47*. https://doi.org/10.1016/j.addma.2021.102273.

4.	Porrelli, D., Abrami, M., Pelizzo, P., Formentin, C., Ratti, C., Turco, G., Grassi, M., Canton, G., Grassi, G., and Murena, L. (2022). Trabecular bone porosity and pore size distribution in osteoporotic patients - A low field nuclear magnetic resonance and microcomputed tomography investigation. J Mech Behav Biomed Mater *125*, 104933. 10.1016/j.jmbbm.2021.104933.

5.	Ulrich, D., van Rietbergen, B., Laib, A., and Ruegsegger, P. (1999). The ability of three-dimensional structural indices to reflect mechanical aspects of trabecular bone. Bone *25*, 55–60. 10.1016/s8756-3282(99)00098-8.

6.	Ding, M., and Hvid, I. (2000). Quantification of age-related changes in the structure model type and trabecular thickness of human tibial cancellous bone. Bone *26*, 291–295. 10.1016/s8756-3282(99)00281-1.
